# Catechol-Loading Nanofibrous Membranes for Eco-Friendly Iron Nutrition of Plants

**DOI:** 10.3390/nano9091315

**Published:** 2019-09-14

**Authors:** Fabrizio De Cesare, Fabrizio Pietrini, Massimo Zacchini, Giuseppe Scarascia Mugnozza, Antonella Macagnano

**Affiliations:** 1 Department for Innovation in Biological, Agro-food and Forest Systems–University of Tuscia (DIBAF), Via S. Camillo de Lellis, 01100 Viterbo, Italy; gscaras@unitus.it (G.S.M.); antonella.macagnano@cnr.it (A.M.); 2 Institute of Atmospheric Pollution Research—National Research Council (IIA-CNR), Research Area of Rome 1, Via Salaria km 29,300, 00016 Monterotondo, Italy; 3 Research Institute on Terrestrial Ecosystems—National Research Council (IRET-CNR), Research Area of Rome 1, Via Salaria km 29,300, 00016 Monterotondo, Italy; fabrizio.pietrini@cnr.it (F.P.); massimo.zacchini@cnr.it (M.Z.)

**Keywords:** nanofibrous membranes, electrospinning, plant nutrition, catechol, Fe-chelating agents, biostimulants, duckweeds, *Lemna minor* L.

## Abstract

Modern agriculture requires more efficient and low-impact products and formulations than traditional agrochemicals to improve crop yields. Iron is a micronutrient essential for plant growth and photosynthesis, but it is mostly present in insoluble forms in ecosystems so that it is often limiting for plants. This study was aimed at combining natural strategies and biodegradable nanostructured materials to create environmentally friendly and low-toxic bioactive products capable of both supplying iron to Fe-deficient plants and reducing the impact of agricultural products on the environment. Consequently, free-standing electrospun nanofibrous polycaprolactone/polyhydroxybutyrate thin membranes loaded with catechol (CL-NMs) as an iron-chelating natural agent (at two concentrations) were fabricated on purpose to mobilize Fe from insoluble forms and transfer it to duckweed (*Lemna minor* L.) plants. The effectiveness of CL-NMs in providing iron to Fe-deficient plants, upon catechol release, tested in duckweeds grown for 4 days under controlled hydroponic conditions, displayed temporal variations in both photosynthetic efficiency and biometric parameters measured by chlorophyll fluorescence and growth imaging. Duckweeds supplied with CL-NMs hosting higher catechol concentrations recovered most of the physiological and growth performances previously impaired by Fe limitation. The absence of short-term toxicity of these materials on duckweeds also proved the low impact on ecosystems of these products.

## 1. Introduction

Modern agriculture has been recently solicited to both improve crop yields and reduce the impact on environments and natural resources. Traditional approaches based on the large use of synthetic chemicals to control plant nutrient deficiencies and diseases have caused soil degradation, environmental pollution, and risks to human health [1], while level rates of yield growth for most crops have been decelerating at the global level [2]. Iron is essential for plants to improve both crop productivity and produce quality, based on human requirements [3], because it is crucial in plant electron transport systems of mitochondria and chloroplasts, where Fe is also a pivotal component of the photosynthetic system [4,5]. Iron is the most abundant metal on the Earth, but in diverse environments, it changes the state of oxidation so that both the various chemical species of iron and the relative amounts depend on the environmental conditions [6,7]. In oxic conditions, iron is mostly present in insoluble ferric hydroxide compounds especially at physiological pH (10^−18^ M for ferric hydroxide solubility at pH 7.4). Hence, iron bioavailability is scarce, and the absorption of this essential element is consequently difficult for both plants and microorganisms living in aquatic environments and in soil so that plants often suffer for iron deficiency [8], with a consequent decrease of food quality [3,9]. Hence, iron is traditionally supplied to plants by farmers employing Fe fertilizers, where Fe is present in chelates (Fe-EDTA), which are applied mostly in foliar spraying but also in solutions to soil. The use of such formulations with Fe-chelates, however, is expensive and moreover these compounds can be easily washed away from leaves by rainfalls, if not promptly absorbed by plants, and leached out from soils because of their high water solubility and mobility, thus causing groundwater and surface water pollution [10]. Therefore, there is a current demand in agriculture for more efficient and effective formulations but also cheaper and more environmentally friendly products than the present fertilizers. To achieve these targets, natural strategies (as bioactive agents) and eco-friendly materials (as carriers) are alternative solutions in controlling plant nutrient deficiencies and diseases.

In natural ecosystems, both microorganisms and plants have evolved various strategies and mechanisms to get Fe from the surrounding environments to match their nutrient demand and prevent its deficiency. One of these strategies is based on the release of iron chelating organic compounds. A wide variety of molecules from diverse chemical classes has been employed or created on purpose by different organisms and even by a single organism, aimed at mining Fe^3+^ from insoluble sources. In the so called Strategy I (nongraminaceous) plants, additional to the acidification of the surrounding environment, the species also uses several classes of compounds, from organic acids (OAs) to phenolic compounds (PCs), that are released as part of exudates from roots outwards to chelate iron with different capacities [11,12,13]. Differently, in Strategy II (graminaceous) plants, highly efficient organic compounds called phytosiderophores (PSs) (siderophores, in the case of microbes (MSs)) are extruded from roots to act as scavengers for Fe^3+^ [14,15,16]. Therefore, the employment of bioactive and biological agents capable of chelating iron (OAs, PCs, or PSs/MSs) could be useful in creating a novel type of iron-supplying products for plants to be applied in agriculture, plant nurseries, and gardening, aimed at being more efficient, effective, and environmentally friendly.

Efficiency in such products can be linked to both the chemical features of the molecules employed and in the capacity of carriers possibly utilized to slowly release bioactive agents in the external medium to enable rapid absorption by the roots, thus preventing possible losses due to reduced bioavailability by adsorption, run-off, and leaching. Some slow/controlled release products based on chemical fertilizers are already commercially available, but they are expensive and often employ plastic coating materials that further contribute to ecosystem pollution [17]. Alternatively, nutrients have been recently bound to nanomaterials (e.g., chitosan) to be supplied to plants [18]. However, a similar strategy cannot be used to provide Fe directly to plants, because the released iron would easily precipitate at typical pH values of both soil and water ecosystems. Moreover, the binding or loading of iron nanoparticles (NPs) to (nano)materials and their following release outwards has not yet been clearly demonstrated to provide Fe directly to plants [19]. It is worth noting that metal NPs are abundant in soils [20], hence the further addition of NPs might cause toxic effects [21]. Finally, the use of Fe-NPs would further increase the exploitation of natural resources to extract iron. Hence, more efficient and eco-friendly materials as carriers for the delivery of Fe-supplying bioactive agents, such as those aforementioned, could be created by employing biodegradable nanomaterials, following what has been recently done in nanomedicine with drug delivery products [22,23].

Electrospinning is a versatile and cheap nanotechnology capable of creating fibers in the range from nano to microscale, as well as nanofibrous fabrics arranged as both thin films and 3D scaffolds, and eventually as both material coatings and free-standing forms for a multitude of applications. Moreover, organic and inorganic polymers (potentially mixed in blends either), biodegradable and non-degradable, as well as natural and synthetic polymers can be employed. These nanofibrous scaffolds can retain a variety of properties (physical, chemical, and biological) that can be further implemented by post-processing functionalization. Additionally, substances of interest (e.g., drugs, effectors, nutrients, antimicrobials, biomolecules) can also be mixed with the carrier polymer solutions before electrospinning or variously linked to functionalized nanofibers and then released with different drug release timings when certain conditions are satisfied [24]. In the last few years, this strategy has been applied for the creation of more efficient fertilizers and pesticides [25]. Hence, electrospinning could also be used to create bioactive nanostructured materials capable of supplying Fe to plants in different contexts through the slow release of Fe-chelating biological agents like those aforementioned. The use of biodegradable organic polymers could further offer the possibility to generate nanofibrous structures with low environmental impact. Polycaprolactone (PCL) is a biodegradable aliphatic polyester that has been employed, based on its hydrophobic properties, for several medical applications [26], and it has also been used to encapsulate compounds in electrospun fibers for drug delivery [27]. Unfortunately, the low melting point value (≈60 °C) makes it useless when employed in environmental applications. Polyhydroxybutyrate (PHB) belongs to polyhydroxyalkanoates, i.e., a class of polyesters produced by several bacteria species mostly as an intracellular storage source of carbon and energy to survive when nutrients become limiting. PHB is a material of interest to create biodegradable, bioderived, biobased, and non-toxic plastics for medicine and other types of applications. Differently from other similar bio-plastics that are water-soluble and also sensitive to water, PHB is insoluble in water and shows some resistance to hydrolytic degradation [28]. Unfortunately, the high crystallinity of PHB makes it rigid and stiff. The use of polymer blends instead of pure polymers is a usual alternative to overcome the weakness of the single polymers and enhance their strengths [29]. PCL exhibits good miscibility with PHB, is ductile, and has a significantly lower melting point than PHB. Thus, nanofibrous fabrics composed of a blend of PCL/PHB can be expected to be soft and mechanically resistant, able to sink, and be poorly affected by sudden changes in temperature, possibly occurring in nutrient treatments to plants, especially in field conditions.

*Lemna minor* L. (duckweed) is the smallest representative of vascular plants, and it is also a model plant frequently used in studies on plant physiology, biochemistry, and ecotoxicology. It is considered, in fact, to be a biological indicator of chemical contamination in aquatic environments, because of its sensitivity to pollutants and other toxic substances [30]. Due to their remarkable ability to absorb and accumulate macro- and micro-nutrients, duckweeds play a crucial ecological role in cycling nutrients in water environments and in reclaiming wastewater [31].

Among the various natural substances capable of chelating iron and employed by organisms to collect it from external environments to fulfill their demand for such micronutrients, catechols are a group of aromatic compounds containing a 1,2-benzenediol moiety that provides them with the capacity of forming stable complexes with a number of di- and trivalent metal ions [32,33,34]. The hydroxyl groups of the catechol ring can strongly coordinate ferric ions because the oxygens in the molecule behave like Lewis bases and hard donor atoms and Fe acts like a hard Lewis acid, thus determining the affinity with iron.

Several distinct organisms (plants and microorganisms as well as mollusks) synthesize and use catechol, although for different purposes [35,36,37,38,39], but always exploiting the Fe-binding capacity of this molecule. Catechol, for example, is part of the phenolic compounds that are the major components of root exudates released in response to iron (Fe) deficiency in Strategy I plants [40,41], and the catechol motif is present in several other soluble phenolic compounds of root exudates, such as coumarins (esculetin, fraxetin, and sideretin) [33,42,43] and flavonoids (quercetin, catechin) [44,45,46], where this structural moiety is specifically devoted to iron binding. A similar strategy is also shared by siderophores, such as catecholates produced by microorganisms [33,44], where a number of catechol motifs are included in the same chemical structure to increase the affinity with iron [36,47,48].

Hence, the aim of this study was to create free-standing thin membranes made of electrospun biodegradable PCL/PHB nanofibrous structures and loading catechol molecules (namely, catechol-loading nanofibrous membranes—CL-NMs) to provide Fe to plants (see Section S1 and Figure S1 in the Supplementary Materials—SM), thus mimicking Strategy I principles. Catechol was preferred as a model and simple iron chelator to phyto- and microbial siderophores because duckweeds are nongraminaceous plants and then they are not expected to have the proper transporters for siderophores. Catechol also has high solubility in water, making it suitable for both hydroponic growth and dissolution in biphasic mixtures of hydrophilic and hydrophobic solvents used to prepare the polymer blend solutions for the fabrication of the bioactive thin membranes by electrospinning. The nanofibrous framework of the scaffold was also preferred to mimic both structurally and functionally the release process of Fe chelators (catechol molecules, in this case) by plant roots. Then, CL-NMs were tested for their effectiveness in providing iron to *L. minor* plants from insoluble sources (FeCl_3_). To test such a capacity of CL-NMs, two catechol concentrations (dose-dependence) and distinct iron treatments (from starvation to optimal concentration) were fixed in hydroponic culturing conditions (see Section S2 in SM). Hydroponic conditions were preferred in this preliminary study to reduce the numerous variables present in tests with real soils. Photosynthetic and biometric parameters were measured in *L. minor* plants undergoing the various treatments. SEM and TEM imaging and analyses were also carried out to investigate the nanostructured materials and the possible effects of the various treatments on plant photosynthetic ultrastructures. The possible successfulness of CL-NMs in supplying Fe to duckweeds would point out the capacity of these plant species of absorbing iron from Fe–catechol complexes, although such findings has never been reported to date in these plants to the best of our knowledge, and would classify the CL-NMs here created as nanostructured biostimulants or nanobiostimulants (NBs) [49].

## 2. Materials and Methods

### 2.1. Nanofibrous Membranes

Non-woven nanofibrous thin membranes (NMs) were produced by electrospinning from a polymer mixture composed of a blend of PCL and PHB (1:0.26, *w*/*w*) added with catechol, as a model iron-chelator (CL-NMs) (see Section S2 in SM, for more details about the procedure used) dissolved at two concentrations (C_1_ = 5 mM and C_2_ = 100 mM). Control NMs were created deprived of catechol (CNMs) (see Section S2 in SM) (Figure S2A). After deposition, CNMs and CL-NMs were removed from the conductive paper discs used to collect them and then cut into 1 × 1 cm (1 cm^2^) pieces (Figure S2A,B). Each squared fragment of the CL-NMs contained approximately 0.50 µmol (C_1_L-NM samples) and 10 µmol (C_2_L-NM samples) catechol. The 1 cm^2^ pieces, irrespective of their catechol content, were then placed on the bottom of the wells of 24-well plates for further incubation with duckweed plants (Figure S1).

Some physical properties of NMs, such as the hydrophobicity (contact angle) and morphology (by AFM and SEM imaging analyses), were analyzed (see Section S3 in SM, for more details).

### 2.2. Plant Material

#### 2.2.1. Plant Material

The genus *Lemna* is often utilized to assess the toxicity of substances to freshwater aquatic plants by growth inhibition tests [50]. Another attractive property of duckweeds is the fast rate of replication so that this genus is extremely suitable for laboratory trials [51,52]. In the present study, duckweed (*Lemna minor* L.) plants were purchased from a specialized company (Anubias, Villanova (BO), Italy) and sterilized [51]. Stock cultures were maintained for one month in a modified Hoagland’s nutrient solution [53], adjusted to about pH 6, in a growth chamber at 25 ± 2 °C, photoperiod 16 h light/8 h dark, and an irradiance of 60 µmol m^−2^ s^−1^, provided by cool white fluorescent lamps (T8 L 58 W/840; Osram AG, München, Germany). Plant cultures were grown under static conditions and subcultured weekly until the beginning of the experiment.

#### 2.2.2. Plant Material Characterization

Some photosynthetic and biometric parameters were used to test the effectiveness of the CL-NMs created here in supplying iron to duckweed plants undergoing various Fe treatments.

In the case of photosynthetic parameters, a non-invasive approach based on chlorophyll fluorescence imaging analysis was used to monitor the same samples over a period of time (see Section S4 in SM, for more details). Specifically, chlorophyll fluorescence parameters such as *F_v_*/*F_m_* (maximum quantum yield of PSII photochemistry), *ΦPSII* (quantum efficiency of PSII), *NPQ* (non-photochemical quenching), and *ETR* (electron transport rate) were measured using saturating light pulses [54,55,56] to estimate photosynthetic activity under different environmental conditions, while total plant chlorophyll content (*Chl*) was monitored by measuring the absorptivity of the frond surface (*Abs*) (see Section S4.1 in SM, for a description of the procedures followed for measuring leaf chlorophyll fluorescence and calculating *ΦPSII*, *ETR*, *NPQ*, and *Chl*).

As concerns the biometric parameters, image analysis was applied to measure the frond number (*FN*), the doubling time (*T_d_*), and the average specific growth rate (*μ_t(0–4)_*) with the aim of testing the effects on *L. minor* plants induced by iron deprivation and its resupply with or without CL-NMs. The procedures utilized for the measurements and calculations of *FN*, *T_d_*, and (*μ_t(0–4)_*) are described in Section S4.2 of SM.

Finally, the effects of the various Fe nutritional treatments on duckweeds were also evaluated by optical and electronic microscopy (TEM) observations of the treated plants. Investigations were focused mostly on the structures and ultrastructures of the plant organelles involved in the photosynthetic process, i.e., the chloroplasts, in both the leaves and roots, where the presence of chloroplasts has also been confirmed by the literature [57] (see Section S3.2.2 in SM, for the procedures followed in TEM observations).

### 2.3. Experimental Design

To assess the capacity of CL-NMs in supplying Fe to duckweeds, plants were pre-treated under Fe-starving conditions and then added with CL-NMs in the presence of insoluble Fe sources. Fully Fe-supplied plants were also tested as controls. To also investigate the singular and combined effects of the various Fe sources and NMs, additional control treatments were also analyzed (see Section S3 in SM, for more details about the experimental design followed) (Figure S3). Duckweeds undergoing the various Fe treatments were incubated for 4 days and samplings were carried out periodically, i.e., at T_0_ and after 24, 48, 72, and 96 h. Each treatment was carried out in six replicates.

### 2.4. Statistics

Normally distributed data were processed by one-way ANOVA, when applicable, using the SPSS 16.0 (Chicago, IL, USA) software tool and a suitable number of replicates per treatment (as indicated in the figures and tables). The statistical significance of the mean data was assessed by the Fisher’s Least Significant Difference (LSD) Test (*p* ≤ 0.05). The Normal probability distribution (Gaussian curve) of the fiber diameters was obtained from the SEM images of the fibers analyzed by Gwyddion© 2.3 software (GNU General Public License) to calculate the fiber size that were then processed by Microsoft Excel© 2011. The distribution analysis of the nanofiber diameters was performed on 150 nanofibers (*n* = 150).

## 3. Results and Discussion

### 3.1. Material Characterization

**Figure 1:**
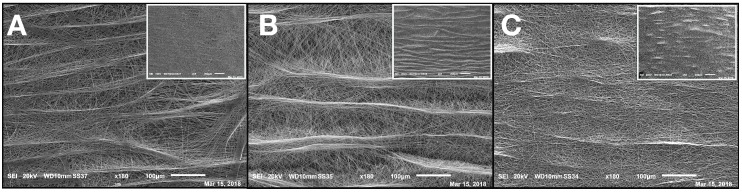
SEM micrographs of the polycaprolactone/polyhydroxybutyrate (PCL/PHB) thin nanomembranes (NMs) after electrospinning deposition and before the incubation with duckweed plants: (**A**) control PCL/PHB nanofibrous scaffolds (CNMs); (**B**) electrospun PCL/PHB NMs loading lower (C_1_) and (**C**) higher (C_2_) catechol concentrations (C_1_L-NMs and C_2_L-NMs, respectively) (magnification ×185); insets = top views of the same nanostructures (magnification ×65). Scale bars in the micrographs correspond to 100 µm (**A**–**C**) and to 200 µm (relative insets).

A single-needle electrospinning procedure was used to generate PCL/PHB nanofibrous membranes in a single step. The depositions were carried out onto filter papers soaked with a PEDOT water solution. Paper was used to facilitate the peeling of the deposited NMs and PEDOT was employed to make the paper more conductive and then more suitable to collect fibers. The PEDOT-soaked paper discs, attached onto the grounded rotating aluminum plate facing the needle tip, successfully collected the ejected fibers within the deposition cone. Despite the presence of a little water fraction (2% *v*/*v*) in the electrospinning PCL/PHB polymer mixture due to the incorporation of the aqueous solution containing catechol molecules, the electrospun liquid jet streams were formed without discontinuity, micro-drops, or nozzle blocks so that fibers could be collected for some hours without interruptions. The various electrospun PCL/PHB NMs, regardless of the catechol loading, were peeled off and analyzed in the morphological and physical features as follows. The distribution of fibers in the thin membranes observed by SEM differed in CNMs, C_1_L-NMs, and C_2_L-NMs nanofabrics. For example, CNMs showed an overall smooth surface at low magnification (×65) (Figure 1A, inset), despite a sort of bundle arrangement of fibers present at the surface and visible at higher magnification only ×185) (Figure 1A).

**Figure 2:**
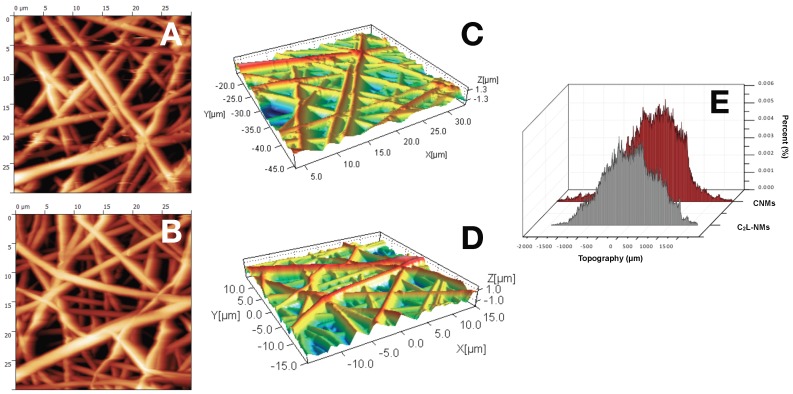
AFM micrographs of 30 × 30 µm scanned areas of PCL/PHB NMs before the incubation with duckweed plants: (**A**) control NMs (CNMs); (**B**) NMs loading higher C_2_ catechol concentration (C_2_L-NMs); (**C**,**D**) 3D-plot of images of (**A**,**B**); (**E**) roughness analysis reporting the percent distribution of peaks and depths in the topography (µm) of the scanned areas of the NMs shown in (**C**,**D**) (positive and negative values of the distribution, respectively).

**Figure 3:**
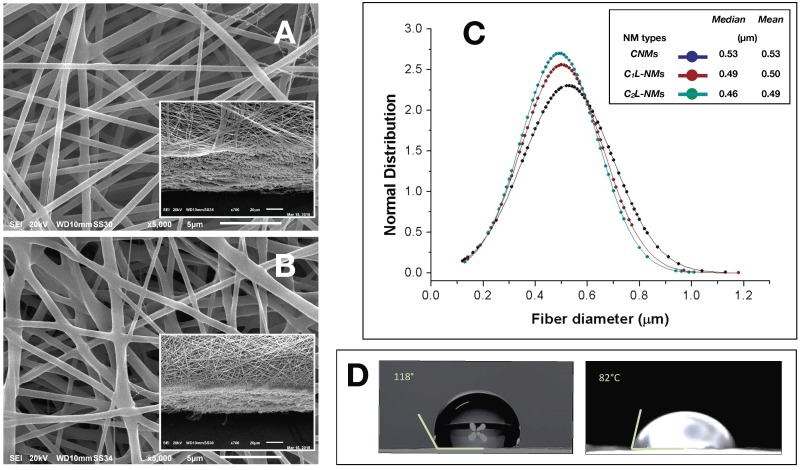
SEM micrographs of CNMs (control) (**A**) and C_2_L-NMs (high concentration catechol loading NMs) (**B**) before the incubation with duckweed plants (magnification ×5000)—insets: tilted cross sections of the same NMs (magnification ×700); (**C**) distribution analysis of the nanofiber diameters present in CNMs, C_1_L-NMs, and C_2_L-NMs (*n* = 150); (**D**) Contact angles of CNMs (left) and C_2_L-NMs (right). Scale bars in the micrographs correspond to 5 µm (**A**,**B**) and 20 µm (relative insets).

The nanofibrous structure appeared as intricate but quite porous (Figure 1A). Differently, the global aspect of C_1_L-NMs (C_1_ = 5 mM catechol initial concentration) at low magnification (×65) looked from the top view like a desert surface with sand ripples shaped by the wind (Figure 1B, inset). At higher magnification (×185), this motif appeared to be derived from a configuration similar to that of CNMs but with larger groups of nanofibers arranged in bundles and generating higher ridges and alternated with wider valleys (Figure 1B). In any case, the C_1_L-NMs framework apparently seemed more porous than that of CNMs (Figure 1A,B). In C_2_L-NMs (C_2_ = 100 mM catechol initial concentration), the whole aspect of the scaffold appeared quite different from the previous ones whatever the magnification used (Figure 1C). The ridges appeared as replaced by isolated crests resulting from fiber coalescence more than from proper bundle aggregation (Figure 1C and inset). The resulting nanofibrous membrane looked much denser and more compact than in the other samples and with visible smaller pores (Figure 1C). The various NMs were further analyzed at higher magnification by both AFM and SEM. The micrographs obtained by AFM from the scanning in the air of 900 µm^2^ NM areas (30 µm × 30 µm) showed no clear difference in the nanofiber morphology of CNMs and C_2_L-NMs samples. A smooth surface, in fact, characterized both the nanofiber types, which appeared deprived of pores notwithstanding the presence of 2% (*v*/*v*) water fraction in the electrospinning polymer mixture (Figure 2A,B). A similar smooth aspect of the nanofiber surfaces was also confirmed in SEM micrographs of the two frameworks (Figure 3A,B). AFM images were also used to measure the roughness of the overall nanofabrics. Figure 2C,D reports 3D-AFM images of both the CNMs and C_2_L-NMs samples, respectively, and the relative topography profile. The selected colors, changing from orange to yellow and to blue, represented the nanofibrous membranes from top to bottom, and highlighted the roughness of the ridges, the entangled structure of the nanofibers, and the void volumes (pores) of the frameworks, respectively. The CNMs sample appeared slightly rougher than the C_2_L-NMs, when measured as *Sa* and *Sq*, since these parameters showed 3% to 5% decrease in C_2_L-NMs (Table 1). Interestingly, the void volume (*Void_vol_*) of C_2_L-NMs showed a substantial decrease relative to CNMs (−16%), thus suggesting that a more compact and less porous fabric resulted from the addition of the high catechol concentration to the polymer blend.

However, since the whole material volume (*Mat_vol_*) was also substantially reduced (−18%), the global volume of C_2_L-NMs was shrunk. This effect was confirmed by the decrease in *Sz* (the sum of the largest peak height value and the largest pit depth value), *Sv* (the maximum pit height—depth), and *Sp* (the highest peak height) (17%, 18%, and 16% decrease, respectively).

The bi-plot reported in Figure 2E confirms the changes of the fabric topography due to the addition of catechol, with a decrease in both the peak and depth distribution in C_2_L-NMs. In addition, the thickness of the electrospun nanofibrous membranes appeared reduced from about 60 µm in CNMs to about 40 µm in C_2_L-NMs (Figure 3A,B insets). Hence, the void ratio (*e* = *V_v_*_/*vs*_, in this case: *Void_vol_* to *Mat_vol_* ratio) of the nanoscaffolds with or without catechol was mostly unchanged in C_2_L-NMs when compared with CNMs material (0.86 vs. 0.84, respectively) so that the calculated porosity (*P* = *e*/*(1 + e)*) was as large as 46% vs. 45%, respectively. These results thus confirmed the observations of C_2_L-NMs s in favor of a more compact overall structure (Figure 1) but without a substantial decrease in the apparent global porosity.

The analysis of the nanofiber distribution based on SEM micrographs of the nanoframeworks showed that both types of the CL-NMs displayed a smaller diameter than the CNMs samples (Figure 3C). Moreover, a dose dependence in the fiber morphology was observed, with the diameter of the fibers decreasing when the catechol concentration loaded increased so that C_2_L-NMs < C_1_L-NMs < CNMs. In detail, the distribution of the C_2_L-NMs nanofibers ranged from 0.130 to 1.010 µm, with a mean diameter of 0.493 ± 0.148 µm; while the C_2_L-NMs nanofibers ranged from 0.140 to 1.180 µm, with a mean diameter of 0.502 ± 0.156 µm; and the CNM nanofibers ranged from 0.120 to 1.130 µm, with a mean diameter of 0.526 ± 0.173 µm (Figure 3C). Therefore, since the global amount of the material deposited was the same in both CNMs and C_2_L-NMs, the reduced dimensions of the nanofiber diameters in C_2_L-NMs caused an increase in the specific surface area that could favor the diffusion of catechol molecules in the external medium. Since the aqueous volumes added to the three polymer solutions were the same, it seemed that the effects on the morphological features of the electrospun nanofibers were independent of the water added but depended on the concentration of catechol in the polymer blend, thus suggesting that catechol induced a rearrangement of the polymer fibers. In any case, the presence of the catechol-containing aqueous solution in the PCL/PHB polymer blend seemed to profoundly affect the physical properties of the resulting nanofibrous membranes that changed from hydrophobic to hydrophilic (Figure 3D). Such a deep modification might be related to changes in the morphology of both the single fibers, which appeared smaller in diameter, and the overall surface of the nanostructured fabrics, which became smoother than the control. In addition, modifications of the surface chemical properties of nanofibers cannot be excluded. The wettability of the fibers did not display, in this case, a clear linear dependence on the quantity of the catechol molecules in the polymer blends, as it was observed instead in the nanofiber size aforementioned, because the contact angle of the C_1_L-NMs could not be measured for the inhomogeneity of the sample. The nanofiber arrangement in bundles at the fabric surface of C_1_L-NMs forming high ridges alternating with large valleys was maybe the cause that inhibited the possibility of having a mat surface regular enough to measure the contact angle. The various electrospun NMs, regardless of the catechol loading, were used for duckweed incubations after peeling off from the conductive paper and cutting into 1 × 1 cm pieces.

### 3.2. Effects of Treatments on Plant Physiological and Biometric Parameters

First of all, it is worth noting, as a general result, that in almost all experimental treatments, *F_v_*/*F_m_* values were mostly within the 0.75–0.85 range, which indicated non-stressed conditions in the plants under study [58]. It is noticeable that plants used in this experiment did not show irreversible symptoms of Fe deficiency at the photosynthetic level due to the Fe-free pre-treatment and that our results were in agreement with data from the literature. It has been shown, in fact, that Fe deficiency leads to decreases in light-harvesting pigments, mainly chlorophylls [59], while several reports suggested that a reduction in the *F_v_*/*F_m_* (value <0.75) occurred only with severe Fe deficiency [59]. In the present study, even when the *F_v_*/*F_m_* values were <0.75, no decrease in the chlorophyll content was observed, suggesting that the effect of Fe deficiency was not dramatic.

#### 3.2.1. Nanomembrane Effects

Possible drawbacks, such as iron adsorption and toxicity, due to the presence of the catechol-free nanofibrous PCL/PHB nanomembranes (NMs) on the *L. minor* plants were assessed in separate samples from the other treatments to distinguish the effects caused by the bioactive agents from those due to the carrier material utilized, which might combine in the resulting values.

##### Nanomembrane Adsorption

**Figure 4:**
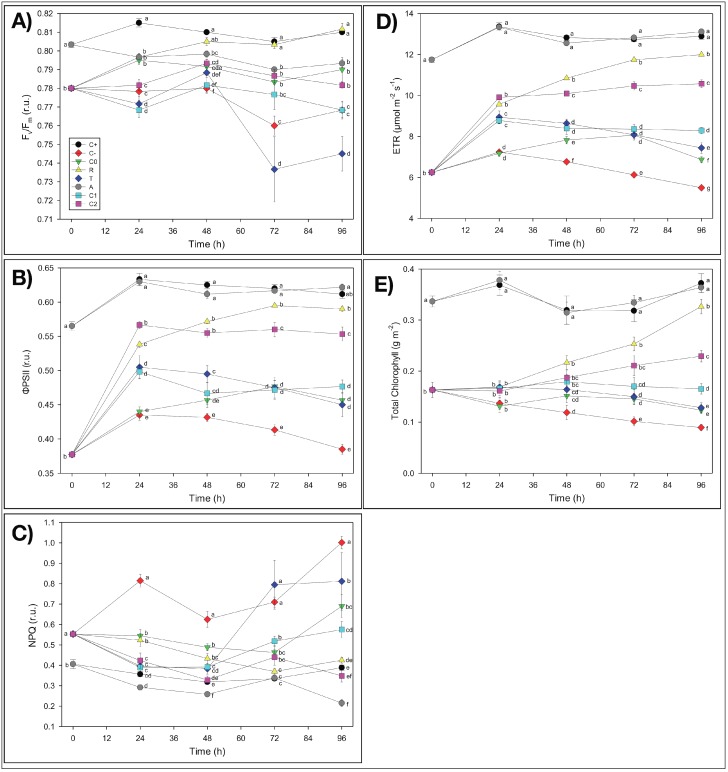
Quantitative analyses of the chlorophyll fluorescence parameters measured in *Lemna minor* fronds treated with different nutrient solutions and electrospun NMs over a 96 h incubation period: (**A**) *F_v_*/*F_m_* = maximum quantum yield of PSII photochemistry; (**B**) *ΦPSII* = quantum efficiency of PSII photochemistry; (**C**) *NPQ* = non-photochemical quenching; (**D**) electron transport rate (*ETR*); (**E**) total chlorophyll content. Check Section S2 in the SM for the explanation of acronyms here associated with graphical symbols as well as for the description of the various treatments and incubation details; and Section S3 for the description of the various parameters tested and procedures used to measure them. Black circle: C^+^; Red diamond: C^−^; Green triangle: C_0_; Yellow triangle: R; Blue diamond: T; Grey circle: A; Cyan square: C_1_L-NMs (C_1_ = 5 mM catechol); Pink square: C_2_L-NMs (C_2_ = 100 mM catechol). Data points and vertical bars represent means (*n* = 6) ± S.E., respectively. One-way ANOVA was applied to each photosynthetic parameter; different letters indicate significant differences between treatments at each incubation period, according to the LSD test (*p* ≤ 0.05).

The possible capacity of NMs to adsorb soluble iron (Fe-EDTA) (A treatment) was assessed indirectly by evaluating possible Fe-deficiency effects on the physiological and biometric parameters induced by Fe binding on NMs from the growth solution during the incubation that might reduce the amount of Fe bioavailable for plant absorption. The duckweed plants treated with pieces of the pristine nanofibrous fabrics (i.e., without catechol) as large as those used for the other treatments did not show any apparent Fe deficiency induced by NMs. The biometric parameters of the A-treated samples were comparable with the control C^+^ as concerns the frond number (*FN*) and even better than C^+^ as concerns both *T_d_* and *μ_t(0–4)_*,. It is worth noting that *T_d_* measures the time required for the frond number to double in value, and since the replication of cell structures and organs requires energy to be spent, only healthy plants can afford to double, thus indicating the degree of stress undergone by plants. As a result, the smaller the doubling time value, the faster the growth is and thus the plant is healthy (low stress). Hence, these results demonstrated that the growth of A-treated plants was even stimulated by the presence of NMs (Table 2). The reasons for such stimulation are still unknown, but the possible concentration of some nutrients by NMs favoring plant nourishment could not be excluded. Besides, these findings were further confirmed by the analyses of possible chlorosis effects in *L. minor* plants and of the chlorophyll fluorescence imaging. At the end of the four-day incubation period, in fact, duckweeds did not show any chlorosis symptoms induced by the reduction of available Fe (Figure S5a,f). As concerns the possible temporal changes of the photosynthetic parameters of duckweed plants measured by fluorescence imaging analyses, the only presence of NMs in the growth medium showed no apparent adverse effects on the photosynthetic activity (*ΦPSII*) and chlorophyll content (*Chl*) of *L. minor* plants with respect to the control (C^+^), with no significant symptoms of nutrient deficiency, consequent to possible Fe adsorption (Figure 4 and Figure S1). On the contrary, even an improvement (as hypothesized for the biometric parameters) as concerns *NPQ* values, which were lower than those of C^+^ (−45%), was observed (Figure 4C). Therefore, the apparent lack of any inhibition by NMs of both the biometric and photosynthetic parameters of duckweeds pointed out that the electrospun thin membranes did not induce iron limitation by removing Fe^3+^ ions from the nutrient solution by adsorption.

##### Nanomembrane Toxicity

Possible toxic effects due to the NMs added to duckweed plants during the 96-h incubation after T_0_ (T treatment) were also assessed by investigating some biometric and photosynthetic parameters. The presence of NMs did not seem to induce any toxicity in duckweeds. It is worth noting that the proper control for this treatment was C^−^, since the T-treated plants were grown in the total deficiency of Fe while in the presence of NMs. The biometric parameters of the T-treated plants highlighted a recovery relative to the poorest conditions of *C^−^* plants, as concerns *FN*, *T_d_*, and *μ_t(0–4)_* (Table 2), suggesting that some stimulating effect was carried out by the electrospun PCL/PHB NMs on the *L. minor* plants. Such “non-toxic” and even improving behavior of the electrospun NMs on duckweed plant growth, instead of the initially hypothesized inhibitory influence, seemed to confirm the nutrient concentration hypothesis suggested for the A treatment. These biometric results were confirmed by the observations of the visual aspect of duckweeds after 96 h of incubation, which showed reduced chlorosis symptoms in the plants, relative to *C^−^* (Figure S5b,e). Additionally, the analyses of the photosynthetic activity and chlorophyll content of duckweed plants grown in the presence of NMs in the growth medium when iron was absent presented values significantly higher than those of C^−^ plants, thus reducing the Fe-deficiency symptoms and showing no toxic effects of NMs on the photosynthetic apparatus, i.e., additional to the absence of Fe as in C^−^ (Figure 4B–E and Figure S4). It is worth noting that the often-reported toxic effects of nanomaterials on living organisms were not observed in the experimental system under investigation, which was composed of electrospun nanofibrous PCL/PHB scaffolds and *L. minor* plants, at least in the short-term period here utilized.

#### 3.2.2. Iron Deficiency Effects

##### Iron Starvation Effects

Duckweed plants undergoing iron starvation throughout the experiment (C^−^), i.e., both before and after T_0_, highlighted severe alterations showing the lowest values in all of the biometric parameters tested, especially relative to C^+^: *FN* (−19%), *μ_t(0–4)_* (−41%), and *T_d_* (+26%, i.e., retardation of duplication) (Table 2). Iron deficiency in C^−^ also negatively affected the photosynthetic performances of *L. minor* plants relative to the C^+^ control (Figure S4 in SM). The photosynthetic activity of *L. minor* plants was notably altered in all of the parameters tested (*ΦPSII*, *NPQ*, *ETR*, and *Chl* content), so that the values measured in total Fe deprivation (C^−^) after the four-day incubation displayed the most significant difference from C^+^ (−37%, +158%, −57%, and −76%, respectively) (Figure 4B–E). Furthermore, the decrease in the photosynthetic activity caused by the Fe deficiency (Figure 4 and Figure S4) was confirmed by the evident chlorosis symptoms in plants after the 96 h incubation period from T_0_ (Figure S5a,b), although these dramatic conditions did not cause a reduction of the *F_v_*/*F_m_* values under 0.75.

##### Iron Scarcity Effects

The addition of iron in the insoluble form (FeCl_3_) to duckweed plants previously starved for iron (Fe-free pre-treatment) *(C_0_)* showed a slight attenuation of the negative effects on the growth of *L. minor* plants observed in C^−^-treated plants, with only a partial (not significant) recovery of the biometric parameters (Table 2). Such improvements indicated that *L. minor* plants after 10 days of Fe-free growth were still preserving their resilience because they were capable of recovering the main functions even when a minimal amount of Fe was available in the medium. Similarly, a slight recovery was also observed in the temporal values of the photosynthetic parameters of C_0_ relative to C^−^: *ΦPSII*, +19%; *NPQ*, −31%; *ETR*, +25%, and *Chl*, +38% (Figure 4D,E and Figure S4). These results were also confirmed by the slight reduction of the chlorosis effects observed in C^−^ (Figure S5b,c).

#### 3.2.3. Iron Resupply Effects

##### Resupply with Soluble Iron

The resupply of iron in the soluble form (Fe(EDTA)) allowed duckweed plants previously grown in the absence of Fe (R treatment) to remarkably recover plant growth, relative to C^−^. This treatment induced a strong significant (*p* < 0.05) improvement of all biometric parameters: *FN* (the highest values), *μ_t(0–4)_*, and *T_d_* (Table 2). The *R* treatment promoted the almost complete recovery of the photosynthetic parameters in duckweed plants after 96 h of incubation, thus approaching C^+^ values with no significant difference, in some cases (Figure 4 and Figure S4). Such improved photosynthetic activity of *L. minor* plants almost cancelled the chlorosis symptoms induced by the previous Fe starvation and still present in Fe-starved C^−^ plants (Figure S5b,d). The results of the R treatment highlighted that duckweed plants were not altered at the physiological level by the 10 days of Fe-free pre-treatment since these characteristics were considerably recovered by the Fe(EDTA) addition to these plants. Our results are in agreement with published papers, where many authors reported that resupplying iron to Fe-deficient plants restores, in a few days, many plant functions, such as chlorophyll concentration and photosynthetic activity, in several plant species [60].

##### Iron Resupply Mediated by CL-NMs

Based on the aforementioned absence of any negative interference by NMs due to nutrient adsorption or toxicity on the physiology and growth of duckweed plants, the effects by CL-NMs on *L. minor* could be reasonably ascribed to the catechol molecules loaded in the nanofibrous fabrics.

The addition of catechol-loading nanofibrous PCL/PHB membranes (CL-NMs) containing catechol and FeCl_3_ to previously Fe-starved duckweed plants showed different trends and results depending on the concentrations of the active compound in the nanofibers. As concerns the biometric parameters, the catechol-mediated iron resupply enabled duckweeds to recover plant growth performances, thus approaching the Fe-soluble supplied treatments (C^+^, A, and R). It is worth noting that C_2_L-NMs *μ_t(0–4)_* was the highest and was significantly greater than C^+^, thus demonstrating a good physiological status of these plants induced by the CL-NMs added, especially if considered in terms of recovery from Fe starvation (C^−^). A detailed analysis of the photosynthetic efficiency showed that such a recovery in CL-NMs was observed in all of the photosynthetic parameters tested except for *F_v_*/*F_m_* (Figure 4). Similarly, CL-NMs-treated duckweeds showed improved results also relative to C_0_-treated plants, where the same source of Fe was supplied in the absence of CL-NMs, with the only exception for *F_v_*/*F_m_*. In detail, the highest performances were observed in C_2_L-NMs, as concerns *ΦPSII*, *ETR*, and *Chl* (+21%, +54%, and +86%, respectively) (Figure 4B–E). It is worth noting that higher recoveries also calculated relative to C^+^ were detected in *ΦPSII*, *ETR*, and *Chl* of C_2_L-NMs (90%, 82%, and 62% of C^+^ values, respectively), and the values of NPQ were even slightly better than in C^+^ (Figure 4C). However, a remaining overall deviation of about −12% (only −2% including the biometric parameters) was present yet, relative to C^+^, so that some chlorosis symptoms were still observed in the C_2_L-NMs plants (Figure S5). In any case, the extent of C_2_L-NMs recovery was exceeded only by the supply of Fe in the promptly bioavailable soluble forms (Fe-EDTA), like in the R treatment, as also confirmed in Figure S4.

These results indicated that, even at the lowest catechol concentration, the CL-NMs was effective, since these bioactive nanofibrous membranes succeeded in providing an adequate amount of iron to *L. minor* plants to support chlorophyll biosynthesis and consequently, the entire photosynthetic process, thus permitting duckweeds rapid recovery from Fe starvation until approaching the nutrient conditions typical of the fully Fe-supplied control. Specifically, by comparing the two CL-NMs, C_2_L-NM-treated plants showed better results in all of the photosynthetic and the biometric parameters than C_1_L-NMs, with an average improvement of 24% for the photosynthetic parameters and of 26% for the biometric parameters (25% improvement globally), thus demonstrating to be more effective than C_1_L-NMs in supplying Fe to duckweed plants because of the more substantial amount of catechol loaded into the nanofibrous membranes. Hence, the duckweed response to the addition of CL-NMs was dose-dependent, relative to the amount of catechol loaded in the NMs.

**Figure 5:**
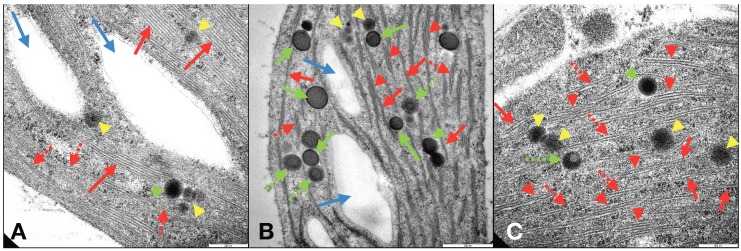
TEM micrographs of *Lemna minor* L. chloroplast ultrastructures in leaf cell cross-sections from plants grown in the presence of total Fe supply (C^+^ control) (**A**), in total Fe deprivation (C^−^) (**B**), and in the presence of C_2_L-NMs and insoluble iron (FeCl_3_). Graphical arrows and arrowheads in the micrographs represent the following: (**A**) light blue arrows = starch granules; solid red arrows = stacked thylakoids in grana; dotted red arrows = single thylakoid stromal lamellae; yellow arrowheads = Type2 plastoglobules (PGs); green arrowheads = Type1 PGs. (**B**) light blue arrows = starch granules; solid red arrows = double thylakoid stromal lamellae; dotted red arrows = grana composed of three stacked thylakoids; red arrowheads = grana composed of ≥four stacked thylakoids; yellow arrowheads = Type2 PGs; green arrowheads = Type1 PGs; solid green arrows = Type3 PGs; dotted green arrows = Type4 PGs. (**C**) solid red arrows = single thylakoid stromal lamellae; dotted red arrows = double thylakoid stromal lamellae; red arrowheads = grana composed of ≥three stacked thylakoids; yellow arrowheads = Type2 PGs; green arrowhead = Type1 PG; dotted green arrow = Type3 PG. Scale bars in the micrographs correspond to 200 nm (**A**,**C**) and 500 nm (**B**).

The improvements observed in duckweeds following the application of CL-NMs and FeCl_3_ to *L. minor* plants were confirmed by the modifications induced in the structures and ultrastructures of duckweeds grown in total Fe-deficient conditions (similar to those in C^−^). Investigations by optical microscope and TEM of C_2_L-NM-treated plant leaves and roots showed a chloroplast abundance and distribution similar to those of C^+^-treated plants (Figures S6a–c and S8a–c) and different from those of C^−^-treated plants (Figure S7A,B). TEM micrographs of the internal membrane organization of the chloroplasts of leaves from C_2_L-NM-treated *L. minor* plants displayed the presence of a diffused membrane system in plant leaf chloroplasts from C_2_L-NMs similar to that of the optimal Fe supply conditions (C^+^) (≤21% of grana stacks and 20% of stromal lamellae relative to the total thylakoid membranes) [61] and different from that of Fe-starved plants (C^−^), as reported in the literature (Figure 5A–C, red arrows) [62]. In C^−^-treated plants, in fact, a residual presence of immature grana composed of two to four thylakoids embedded in the stroma (Figure 5B, red arrows) was observed, further reduced to hardly visible faint lamellae immersed in the granular and electron-dense stroma (Figure S7B) (see also SM, for more details).

Other pieces of evidence on the ultrastructural effects induced by the addition of CL-NMs to Fe-deficient (insoluble Fe) duckweeds were the presence, additional to starch granules, of some circular electron-dense structures visible by TEM in leaf chloroplasts and named plastoglobules (PGs) (Figure 5C). PGs are lipoprotein bodies also containing tocopherols (vitamin E) and other isoprenoid lipids and related metabolites that are localized in chloroplasts and other plastids and are related to lipid storage and metabolism and to various types of stress responses. Depending on the stressing cause, the presence and number of PGs vary, as well as their association with and connection to thylakoid membranes [63]. PGs have been related to stresses, like high light, Fe-deficiency, salt concentration, and heavy metals [64,65,66]. In the present study, PGs were present in both leaf and root cells of plants from all treatments and displayed different types of structures assorted distinctly between the treatments and might be related to diverse functions or degree of maturation (Figure 5A–C green arrows and yellow arrowheads) [63,67]. Although PGs have been widely studied, the presence of distinct types of PGs in the same plant species and contemporary in the same plant leaves and even in the same chloroplast seemed to be a piece of unique evidence to date to the best of our knowledge. In any case, the heavily stressing growth condition induced by Fe deprivation in C^−^ plants clearly affected the ultrastructural organization of the photosynthetic organelles, which were profoundly altered in the thylakoid membrane distribution and organization. It appears that the modifications (dismantling) in the membrane organization from C^+^ to CL-NMs to C^−^ were closely related to the number (and maybe the function) of PGs in the chloroplasts. Such modifications might be related to the role of PGs in the reconstitution and repair of the thylakoid membranes upon stressing events [68].

Regardless of the performances of the CL-NMs in supplying Fe to duckweeds, it is worthy to note that this study provided pieces of evidence, for the first time to our knowledge, that *L. minor* plants are capable of using catechol to absorb Fe for their nutrition and metabolic activities. Duckweeds are known generally to absorb iron from the outer environment by a constitutive system for transmembrane transfer of electrons (using cytosolic NAD(P)H) aimed at reducing extracellular acceptors like Fe^3+^ (reduced to Fe^2+^). However, this Fe-transport system is also able to get iron from Fe^3+^ chelates (Fe-EDTA) so that the chelate reduction rate in Fe deficiency is even higher than that of the free ions [69]. Catechol is known to be used by nongraminaceous plants to chelate iron present in the external environment to facilitate its uptake by plants. However, duckweeds have not been reported to date to have the capacity to use this molecule with this aim. *Lemna* spp. have also been reported to absorb even ferrichrome, a hydroxamate siderophore synthesized and released by bacteria, through the plasmalemma [70], similar to other Strategy I plants [71], may be by mechanisms other than those typical of these plants (e.g., endocytosis) [71,72].

### 3.3. Environmental Significance

The novelty of this study is that a natural strategy of iron nutrition typical of plants based on the release of organic Fe chelators was integrated with a nanofibrous fabric as the carrier of such bioactive natural molecules to mimic the release from the plant roots (mostly root hairs) of such Fe-mobilizing compounds aimed at providing Fe to plants. Fibrous PCL/PHB nanostructured fabrics loaded with catechol as a natural Fe-chelating agent, which is usually synthesized and released by nongraminaceous plants to improve Fe uptake, were utilized in this study to supply iron to Fe-starved duckweeds. The artificial NMs here fabricated were characterized by a hydrophilic surface and an extensive specific surface area providing a large diffusion surface for the release of the iron-chelating molecules previously loaded in the nanofiber polymer blend. In the short-term incubation of the study, the free-standing catechol-loading nanomembranes (CL-NMs) created here were effective in rapidly mobilizing iron from insoluble sources (FeCl_3_) and supplying it to Fe-starved duckweed plants. The investigations on *L. minor* plants showed, in fact, that upon addition of CL-NMs to Fe-starved duckweeds, several improvements occurred in the plants, such as enhanced chlorophyll synthesis (molecular level), greater photosynthetic activity (physiological and metabolic level), ultrastructural reorganization of the photosynthetic organelles (structural level), and reactivation and stimulation of plant growth (biometric level), so that all these parameters previously impaired by the Fe limitation were successfully recovered to a large extent or totally. The recovery observed following the addition of CL-NMs was even greater at the highest catechol concentration than that measured upon addition of promptly available Fe (Fe(EDTA) in R treatments), in some biometric parameters, and it approached the values of the optimal Fe-supplied plants (C^+^). Therefore, based on the features of the NMs generated here (employing a natural Fe-chelating agent) and the findings (improvement of Fe nutrition in plants) obtained, the final products can be reasonably classified as proper biostimulants. Specifically, because of the presence of nanomaterials in the composition of such products, the latter could be named as nanobiostimulants (NBs).

In addition, with the aim of obtaining final fully eco-friendly products, biodegradable polymers, namely a blend of PCL/PHB, were employed to create on purpose Fe-supplying electrospun nanofibrous membranes loading catechol as a natural Fe-chelating agent (CL-NMs). Such an approach was also proven to be successful in generating low-toxicity products supporting plant growth, since no negative effects in the photosynthetic and biometric parameters tested were observed in *L. minor* plants treated with only NMs.

It is worth noting that additionally to the findings aforementioned concerning the effectiveness of the created NBs in supporting the Fe nutrition of plants and their inherent low impact, further valuable advantages consequent to the use of these NBs in plant culturing (in agriculture, nurseries, and gardening) could be the following: (i) The use of eco-friendly biodegradable polymer materials as carriers could replace the plastic materials commonly used in the commercial slow-release fertilizers. (ii) The use of CL-NMs employing a natural Fe-chelating agent, such as catechol, could replace the use of synthetic chemicals (Fe chelates like Fe(EDTA)), typically utilized in traditional fertilizers, thus reducing the global impact on the environment and health. (iii) The encapsulation of catechol molecules into the nanofibers since causing the slow release of these bioactive agents in the external medium, the mobilization of Fe from insoluble natural Fe sources and its rapid absorption by organisms would prevent the leaching of Fe chelates and the possible pollution of water sources as happens with synthetic chemical Fe chelates (Fe(EDTA)), and would also reduce the global amounts of fertilizers. (iv) The employment of Fe-chelating compounds, like catechol, in the CL-NMs to supply iron to plants was aimed at mobilizing Fe from the natural insoluble sources present in the environment surrounding the plants (e.g., natural stocks in soil and sediments). Oppositely, the use of synthetically preformed Fe chelates would require the previous mining of Fe from some natural resources to then be bound to the synthetic Fe chelator (or in the use of Fe-NPs). (v) Finally, the utilization of reusable organic chelating agents, like catechol (until degradation), that after Fe transfer to plant cells can be reused for the same activity and purpose would prolong the Fe-supplying effect of the NBs created (CL-NMs) here for a longer time relative to that of other Fe supplying products (e.g., Fe-NPs, sulphur, FeS), thus making these NBs more efficient.

## 4. Conclusions

In conclusion, the findings reported here demonstrated that the combination of natural strategies and biodegradable materials could result in a successful approach to create novel and valuable low-impact and sustainable nanobiostimulants capable of overcoming the limitations caused by the scarce bioavailability of Fe for plants in hydroponic cultivations and, potentially, in croplands. The application of such bioactive products in agriculture (but also in nurseries and gardening) could be powerful in improving crop production and consequently contributing in fulfilling the demand by human populations for healthy and safe foods and environment preservation.

Further studies are necessary to assess whether the effectiveness of the nanofibrous PCL/PHB membranes in releasing catechol depends only on the concentration gradient of this model molecule between the interior of the fibers and the outer solution, or also on the physical properties of the fibers induced by the presence of the specific Fe chelator used.

## Figures and Tables

**Table 1:** Roughness parameters of the electrospun nanofabrics CNMs and C_2_L-NMs. Area = area scanned by AFM; *Mat_vol_* = volume of the material; *Void_vol_* = volume of the voids within the scanned area; *S_a_* = roughness average (i.e., the difference in height of each point compared to the arithmetical mean of the surface), *S_q_* = the root mean square value of ordinate values, *S_z_* = the sum of the largest peak height value and the largest pit depth value, *S_v_* = the absolute value of the height of the largest pit, and *S_p_* = the height of the highest peak.

Parameters	CNMs	C_2_L-NMs	Variation
***Area* (µm^2^)**	900	900	—
*Mat_Vol_*(µm^3^)	2160.7	1776.1	−18%
*Void_Vol_*(µm^3^)	1819.2	1533	−16%
***S_a_* (µm)**	0.542	0.525	−3%
***S_q_* (µm)**	0.678	0.642	−5%
***S_z_* (µm)**	4.388	3.648	−17%
***S_v_* (µm)**	2.378	1.958	−18%
***S_p_* (µm)**	2.011	1.690	−16%

**Table 2:** Growth parameters of *Lemna minor* L. measured after treatments for 96 h (4 days) with different nutrient solutions and nanofibrous products (NMs): *FN* = frond number; *T_d_* = doubling time of the *FN*; *μ_t(0-4)_* = average specific growth rate; C^+^ = fully Fe-supplied duckweed plants; C^−^ = totally Fe-deprived duckweed plants; C_0_ = duckweeds grown in Fe-limiting conditions; *R* = *L. minor* plants resupplied with soluble Fe after an Fe-free pre-treatment; T = plants grown as C^−^ and also incubated with NMs (catechol-free); A = duckweeds grown as C^+^ and also incubated with NMs (catechol-free); C_1_L-NMs = plants as in C_0_ and also incubated with NMs loading catechol at a low concentration (C_1_ = 5 mM catechol); C_2_L-NMs = plants as in C_0_ and also incubated with NMs loading catechol at a high concentration (C_2_ = 100 mM catechol) (check the text for more details). Data are the mean values of six replicates ± standard error (SE). One-way ANOVA was applied, and data followed by different letters in the same row are significantly different (LSD test, *p* < 0.05).

Parameters	C^+^	C^−^	C_0_	R	T	A	C_1_L-NMs	C_2_L-NMs
***FN* (n_t4_)**	6.8 ± 0.1 ^ab^	5.5 ± 0.6 ^b^	7.5 ± 1.0 ^ab^	8.1 ± 0.9 ^a^	6.5 ± 1.9 ^ab^	7.3 ± 0.5 ^ab^	6.5 ± 1.9 ^ab^	7.3 ± 0.5 ^ab^
*T_d_*(days)	2.20 ± 0.04 ^de^	2.78 ± 0.12 ^a^	2.51 ± 0.07 ^ab^	2.21 ± 0.07 ^cde^	2.41 ± 0.03 ^bcd^	2.01 ± 0.06 ^e^	2.41 ± 0.03 ^bcd^	2.01 ± 0.06 ^e^
*µ_t(0-4)_*(days^−1^)	0.22 ± 0.01 ^b^	0.13 ± 0.02 ^d^	0.16 ± 0.01 ^cd^	0.22 ± 0.02^b^	0.18 ± 0.01 ^bc^	0.27 ± 0.02 ^a^	0.18 ± 0.01 ^bc^	0.27 ± 0.02 ^a^

## Supplementary Materials

The following are available online at https://www.mdpi.com/2079-4991/9/9/1315/s1, Figure S1. Sketch of the experimental plan describing the preparation of the catechol-loaded nanomembranes (CL-NMs), Figure S2. Electrospun nanomembranes (NMs) (irrespective of the catechol loading) after deposition onto the PEDOT-soaked paper attached to an aluminum disk (A); (B) NMs peeled off the mentioned supports; (C) NMs after cutting into 1-cm^2^ pieces, Figure S3. Sketch of the experimental plan describing the various Fe-treatments of duckweed plants to test the performances of the distinct catechol-loaded nanomembranes (CL-NMs), Figure S4. Time courses of chlorophyll fluorescence parameters in *Lemna minor* L. fronds treated with different nutrient solutions and electrospun nanofibrous products for (4 days) after T_0_. Images of the duckweed plants (captured by an Imaging-PAM M-series system) at actinic illumination of 60 μmol photons m^−2^ s^−1^ after 0, 24, 48, 72 and 96 h of treatment from T_0_ showing: (i) *Fv*/*Fm* = maximum quantum yield of PSII photochemistry in a dark-adapted leaf, (ii) *ΦPSII* = quantum efficiency of PSII photochemistry and (iii) *NPQ* = non-photochemical quenching measured at steady-state. The arbitrary color code depicted at the bottom of each image ranges from 0.000 (black) to 1.000 (pink). Acronyms used for the various treatments are the same as reported in Figure 4 (main text). The image also highlights the presence of an uneven spatial distribution of the photosynthetic efficiency caused by distinct treatments of iron deprivation that can be frequently observed both in different leaves of the same sample and even within each leaf, Figure S5. Pictures of duckweeds fronds captured before (T_0_) and after (T4d) various treatments: (a) C^+^ = 6 μM Fe(EDTA) for both pre-treatment and treatment; (b) C^−^ = no Fe for both pre-treatment and treatment; (c) C_0_ = no Fe (pre-treatment) + 6 μM FeCl_3_; (d) R = no Fe (pre-treatment) + 6 μM Fe(EDTA); (e) T = no Fe for both pre-treatment and treatment + catechol-free nanomembranes (CNMs); (f) A = 6 μM Fe(EDTA) for both pre-treatment and treatment + CNMs; g) C_1_LMNs = no Fe (pre-treatment) + 6 μM FeCl_3_ + C_1_L-NMs (C_1_ = 5 mM catechol); h) C_2_L-NMs = no Fe (pre-treatment) 6 μM FeCl_3_ + C_2_L-NMs (C_2_ = 100 mM catechol), Figure S6. Optical microscope images and TEM micrographs of *Lemna minor* L. structures of leaves (A) and roots (B,C,D) from C^+^ plants: (A) cross section of a mesophyll leaf observed by TEM; (B) and (C) = longitudinal and transversal cross sections, respectively, from duckweed roots observed by optical microscope (×100); red arrows treachery structures; orange arrows chloroplasts in root cells surrounding the treachery structures; (D) ultrastructural features of a chloroplast from a C^+^ root cell: solid red arrows = double thylakoid stromal lamellae; dotted red arrows = ≥3 thylakoid stromal lamellae; green arrows = Type1 PGs; inset = TEM micrograph of a longitudinal cross section of a C^+^ root showing chloroplasts with starch granules (light blue arrowheads) and the treachery structures (red arrowhead), Figure S7. TEM micrographs of *Lemna minor* L. structures of leaves (A) and roots (B,C) from Fe-deprived C^−^ plants: (A) TEM micrograph of a mesophyll cell showing chloroplasts with starch granules (pale arrows and inset, light blue arrowheads); solid and dotted green arrows = Type1 and Type2 PGs, respectively; pink arrows = faint thylakoids in stromal lamellae; (B) cross section of a duckweed root cell showing starch granules (light blue arrowheads); (C) ultrastructural features of a chloroplast from a C^−^ root cell: red arrowheads = single thylakoid stromal lamellae; solid red arrows = triple thylakoid stromal lamellae; dotted red arrows = ≥3 stacked thylakoids in immature grana; green arrows = Type1 PGs; green arrowheads = Type2 PGs; light blue arrowheads = starch granules, Figure S8. Optical microscope images and TEM micrographs of *Lemna minor* L. structures from plants treated with C_2_L-NMs and FeCl_3_ (A = image from a duckweed leave; b,c,d = images from duckweed roots. (A) TEM micrograph of a mesophyll leaf: light blue arrows = chloroplasts with starch granules; (B,C) longitudinal and transversal cross sections of roots observed by optical microscope (×100): red arrowheads treachery structures; orange arrowheads chloroplasts attached to vacuoles in root cells surrounding the treachery structures; (D) ultrastructural features of a chloroplast from a C_2_L-NM root cell: red arrows = single, double and triple thylakoid stromal lamellae; red arrowheads = ≥3 thylakoid stacked grana; green arrows = Type1 PGs; green arrows = Type3 PGs; inset = TEM micrograph of C_2_L-NM root cells showing chloroplasts without starch granules (light blue arrowheads).
