# Supplementary material for: Catechol-Loading Nanofibrous Membranes for Eco-Friendly Iron Nutrition of Plants
Source: Nanomaterials (Basel). 2019 Sep 14;9(9):1315. doi: 10.3390/nano9091315 (PMC6781093; doi:10.3390/nano9091315)
Supplement: Supplementary file 1 [file nanomaterials-09-01315-s001.pdf]

SUPPLEMENTARY MATERIALS TO

"Catechol-Loading Nanofibrous Membranes for Eco-friendly Iron Nutrition of Plants"

F. De Cesare[ab], F. Pietrini[c], M. Zacchini[c], G. Scarascia Mugnozza[a], A. Macagnano[b]

[a]Department for Innovation in Biological, Agro-food and Forest Systems (DIBAF), University of Tuscia, 01100 Viterbo, Italy
[b]Institute of Atmospheric Pollution Research (IIA), National Research Council (CNR), 00016 Monterotondo (Rome), Italy
[c]Research Institute on Terrestrial Ecosystems (IRET), National Research Council (CNR), 00015 Monterotondo (Rome), Italy

SUPPLEMENTARY MATERIALS

S1 – Aims of the study

As reported in the main text, the main aim of the study consisted, in short, in the preparation of nanofibrous membranes housing catechol molecules (see Section S2) and their addition to duckweed plants grown under hydroponic conditions (see Section S5) to test their capacity of providing Fe to them (Figure S1).

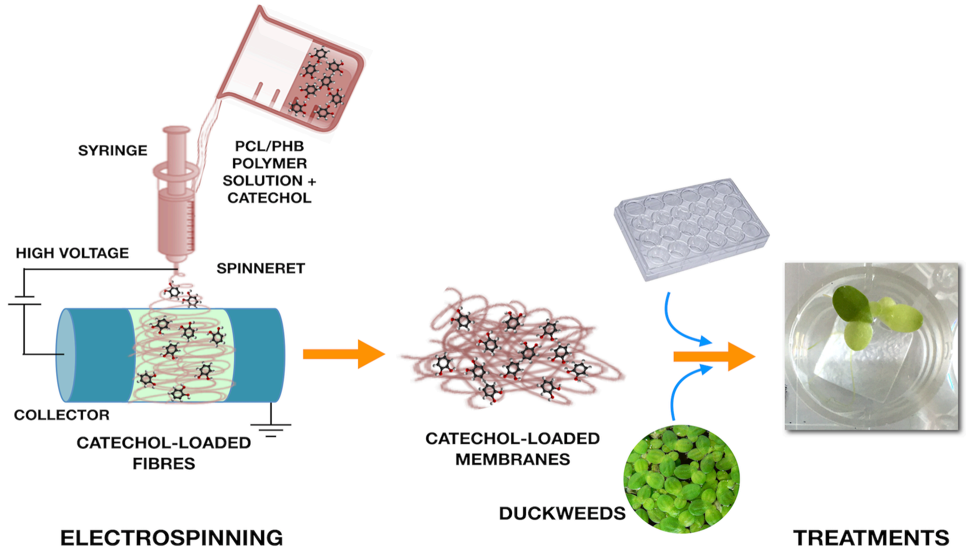

FIGURE S1. Sketch of the experimental plan describing the preparation of the catechol-loaded nanomembranes (CL-NMs)

MATERIALS & METHODS

S2 - Nanofibrous membranes (NMs) fabrication

To create non-woven electrospun nanofibrous thin membranes, 10 mL PHB solution in TFE/CHCl3 (trifluoroethanol/chloroform, 9.9:0.1, v:v) was prepared by solving 0.515 g of powder at 65°C under stirring for 72 h, at least. Similarly, 1.22 g PCL was dissolved in 10 mL TFE/$CHCl_3$ (2:8, v:v) at 60°C for 24 h. Finally, the PHB and PCL solutions were mixed in order to obtain a polymer blend with a mass ratio of 1:0.26 (w:w), respectively. Two catechol solutions were prepared separately by dissolving catechol (purchased from Sigma-Aldrich) in ultrapure water at two concentrations (5 mM and 100 mM). Electrospinning solutions containing catechol at the two concentrations and the PCL/PHB polymer blend were prepared by incorporating 0.10 mL catechol solutions (5 mM or 100 mM) in 5 mL PCL/PHB polymer solution (2% v/v). The distinct samples were then sonicated in an ultrasonic water bath (Branson 1800) for 5 min to obtain homogeneous mixtures. Control electrospinning solutions without catechol were prepared as in the previous case but mixing the same ultrapure water volume to the PCL/PHB polymer solution. Nanofibres were then generated using a home-made electrospinning machine (CNR-IIA, Italy) composed of a high power AC-DC (alternative current-direct current) converter, a high voltage oscillator (100 V) to stabilise the electric field (ranging from 1 to 50 kV) and a syringe pump (Model KDS 200, KD Scientific). The electrospinning machine was placed in a home-made clean-box housing temperature and humidity sensors, and was set up as follows:

4.0-4.5 kV electrostatic DC voltage between the syringe stainless steel needle tip and the grounded collector set at 5 cm distance and perpendicular to the syringe. The single-needle syringe holding the polymer solution was incorporated in the syringe pump working at 1000 µL h^{-1} in feed rate. Nanofibres were collected on a conductive paper disc as follows: 6.5 cm diameter filter papers were soaked with a PEDOT water solution (2% v/v) and let them absorb it for 5 min, then they were oven dried at 60°C and placed on a 7 cm aluminium disc fixed on a clockwise rotating metal collector (600 rpm), and finally exposed to the electrospinning deposition until thin free-standing nanofibrous membranes (*NMs*) were created (for 3 h at about 28°C and 30% RH) (Figure S2).

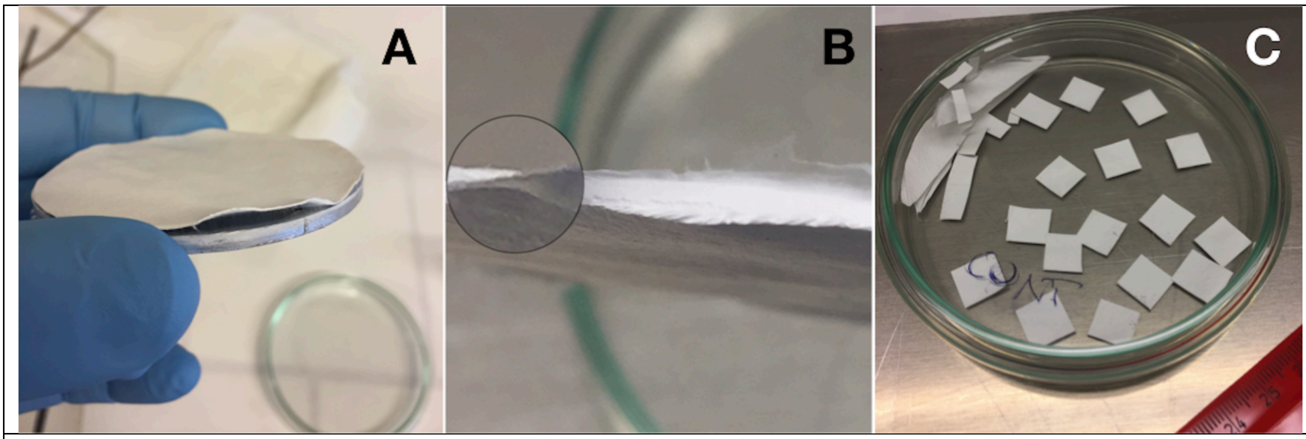

FIGURE S2. Electrospun nanomembranes (NMs) (irrespective of the catechol loading) after deposition onto the PEDOT-soaked paper attached to an aluminium disk (**A**); (**B**) NMs pealed off the mentioned supports; (**C**) NMs after cutting into 1 cm^2 pieces.

S3 - Nanofibrous membranes (*NMs*) characterisation

The various *NMs* were characterised as concerns their hydrophobicity (contact angle) and morphology (AFM and SEM).

S3.1 - Contact angle

The contact angle of the *NMs* was measured from water drops (8 µl) deposited on the PCL/PHB nanofibrous fabrics with or without catechol molecules upon electrospinning deposition. Images were captured by a USB-Digital Microscope (2.0 MPx, DIGIMICROSCOPE). The water contact angle was quantified in the thin membranes after 5 s of water drip by DropSnake© (LBADSA method), a plugin implemented for ImageJ software (open source image processing program).

S3.2 - Imaging analyses

S3.2.1 - AFM Imaging

The AFM (atomic force microscopy) micrographs were taken in tapping mode using a 190Al-G tip, 190 kHz, and 48 N m−1 (Nanosurf AG, Liestal, Switzerland; FlexAFM) on the nanofibrous layers. The roughness of the fibrous layers after AFM scanning was measured using SPIP 6.7.6 software (Image Metrology, Denmark) over image areas of 900 µm2 (30x30 µm). A plane correction process was performed on all of the AFM topographical images. The roughness parameters here analysed within the defined area were the roughness average (i.e. the difference in the height of each point compared to the arithmetical mean of the surface) (Sa), the root mean square value of ordinate values (Sq), the sum of the largest peak height value and the largest pit depth value (Sz), the absolute value of the height of the largest pit (Sv) and the height of the highest peak (Sp).

S3.2.2 - SEM Imaging

The various types of NMs, i.e. CNMs and CL-NMs, to be analysed by SEM were fixed in glutaraldehyde (2.5% v/v) + paraformaldehyde (2% v/v) solved in 0.1 M cacodylate buffer pH 7.2 overninght at 4°C. After cold washings in the same buffer, post-fixation in osmium tetroxide (2% v/v) in cacodylate buffer for 2 h at 4°C was then performed. Then, a cold rinsing was applied to the processed specimens before dehydration in a graded ethanol series following the critical point method with CO_2 in a Balzers Union CPD 020. NMs were finally sputter-coated with gold in a Balzers MED 010 unit before observations by a JEOL JSM 6010LA electron microscope.

S4 – Plant material characterisation

To test the effectiveness of the various Fe-treatments (Section S5) on duckweed plants, several physiological and biometric parameters were measured.

S4.1 - Photosynthetic parameters measurements

Chlorophyll fluorescence analysis has been used for many years as a rapid non-destructive tool to evaluate the state of the photosynthetic apparatus in many plant species. In the last years, with the introduction of imaging instrumentation that maps chlorophyll fluorescence parameters, it has been possible to identify spatial heterogeneity of leaf photosynthetic performance [2,3]. This technique has also been used to assess the effects of several contaminants, including pharmaceuticals [4] and heavy metals [5], on photosynthetic efficiency of *Lemna* plants. Recently, the use of chlorophyll fluorescence imaging has been utilised to investigate the impact of iron deficiency and resupply on the photosynthetic performance of strawberry plants [6]. On the other hand, leaf chlorophyll content is one of the most critical factors in determining photosynthetic potential and primary production [7], and chlorophyll reduction upon Fe-deficiency conditions has also been reported in many studies [8,9]. The chlorophyll fluorescence imaging was then used to monitor the temporal changes in both the photosynthetic efficiency and the chlorophyll content during the overall 14 day-period of treatments of plants, i.e. the 10 day-pre-treatment and the following 4 day-treatments (i.e. at T_0 and after 24, 48, 72 and 96 h of exposure), consequent to the various treatments of plants in the presence or absence of Fe (see Section S5). The F_v/F_m ratio is a parameter frequently used to detect disturbances in the photosynthetic system favoured by several stress, because its decrease indicates a reduction in the photochemical efficiency of PSII and damages to the photosynthetic apparatus [10]. ΦPSII is the most useful parameter to measure the efficiency of PSII photochemistry [11]. It measures the proportion of the light absorbed by chlorophyll associated with PSII that is used in photochemistry and, under laboratory conditions, there is a strong linear relationship between this parameter and the efficiency of carbon fixation. Moreover, ΦPSII can be used to calculate linear electron transport rate (ETR) contributing to represent the plant photosynthetic capacity in vivo [12]. Differently, NPQ is often used as an indicator of the excess-radiant energy dissipation to heat in the PSII antenna complexes [10]. This is a photoprotective process that removes excess excitation energy within chlorophyll-containing complexes and prevents the likelihood of formation of damaging free radicals [13].

S4.1.1 - Photosynthetic efficiency based on chlorophyll fluorescence imaging analysis

The chlorophyll fluorescence was calculated by averaging all data points over the entire surface of the leaf. Briefly, transparent 24-well plates housing *L. minor* plants were placed into the sample plane of a MAXI-Imaging-PAM fluorometer (Walz, Effeltrich, Germany). The plants were firstly dark-adapted for at least 30 min, and then minimal fluorescence (F_0) was measured (0.5 μmol m^{-2} s^{-1} PAR, λ = 450 nm). Maximum fluorescence (F_m) was then measured using a saturation light pulse (2700 μmol m^{-2} s^{-1} PAR, 800 ms duration, λ=450 nm) and the maximum quantum yield of PSII photochemistry (F_v/F_m) was calculated as $(F_m - F_0)/F_m$. Subsequently, the plants were adapted to a light intensity of 60 μmol m^{-2} s^{-1} for at least 10 min to reach a steady-state condition. Immediately after, a saturation light pulse was applied to determine the maximum fluorescence ($F_{m'}$) and steady-state fluorescence (F_s) during the actinic illumination. Saturation light pulse images and values of the chlorophyll fluorescence parameters were captured. Data obtained were used for the calculation of the quantum efficiency of PSII photochemistry (*ΦPSII*) and the non-photochemical quenching (*NPQ*), as described in Pietrini et al. [13]. In Figure S4, F_v/F_m, *ΦPSII* and *NPQ* were reported using a false colour code depicted at the bottom of each image ranging from 0.000 (black) to 1.000 (pink). In the case of *NPQ* reported in the same figure, measures were divided by four to display values < 1.00. The electron transport rate (*ETR*) was calculated as follows:

$$ETR = \Phi PSII \cdot PAR \cdot 0.5 \cdot Abs, \qquad (1)$$

where Abs is the apparent absorptivity of the frond surface, and 0.5 is the fraction of light absorbed by PSII antenna [10,12]. The Abs value was automatically calculated pixel by pixel from the R-(red) and NI-(near infrared) images based on the equation:

$$Abs = 1 - \left({}^{R}/_{NIR}\right), \qquad (2)$$

S4.1.2 - Total chlorophyll content

The absorptivity *(Abs)* image is a measure of the fraction of the incident Red-light which is absorbed by the leaf sample and is calculated using the equation (2). This measurement is based on previous calibration of the MAXI-Imaging-PAM fluorometer aforementioned, which involves appropriate adjustment of the R-intensity (660 nm) with respect to the NIR-intensity (780 nm). The instrument is considered as properly calibrated when the R- and NIR-images of a white piece of paper show similar brightness values. Then R/NIR is close to unity and then *Abs* is close to zero. Any pigment that absorbs Red more than NIR light, i.e. generally all photosynthetically active pigments (mainly chlorophyll a and b), will decrease R with respect to NIR, thus decreasing R/NIR and increasing the derived *Abs*-value. On the other hand, pigments that absorb Red and NIR light similarly, as e.g. necrotic spots, will not cause R/NIR to deviate substantially from unity and thus Abs. The obtained values of *Abs*

were used to calculate the total chlorophyll content (*Chl*) expressed in g *Chl* per m^{-2} of leaf area by applying the modified equation of Evans [14] as follows:

$$Chl(gm^{-2}) = \left[{(Abs \cdot 0.074)}/{(1 - Abs)}\right] \cdot 0.9, \quad (3)$$

where 0.074 is an empirical constant with the dimension of mmol m^{-2}, and 0.9 is a coefficient to convert the chlorophyll content from mmol m^{-2} to g m^{-2}.

S4.2 - Biometric parameters

The biometric parameters were measured at T_0 and after 24, 48, 72 and 96 h of treatment exposure using imaging analysis software (ImageJ, IJ 1.46r, *http://imagej.nih.gov/ij/*). Specifically, T_d and ($\mu_{t(0-4)}$) were calculated based on the total frond number *(FN)*. *FN* was determined in duckweed plants present in each replicate of the various treatments in the presence or absence of Fe. The fronds in each plate well were counted, and their area was recorded preventing possible overlapping. The average specific growth rate ($\mu_{t(0-4)}$), expressed in day^{-1}, based on the total *FN* was calculated over the 4 d-period (96 h) of treatment *(*$\mu_{t(0-4)}$*)* from the following equation:

$$\mu_{t(0-4)} = \left({(ln(FN_{t4}) - ln(FN_{t0}))}/{(t4 - t0)}\right), \quad (4)$$

while the doubling time *(*T_d*)*, expressed in days, of the *FN* was determined according to the Test No. 221 of the OECD Guidelines for the Testing of Chemicals, Section 2 [15] from the following equation:

$$T_d = {ln2}/{\mu_{t(0-4)}} \quad (5)$$

S4.3 - Imaging analyses

S4.3.1 - Optical Imaging

Plant samples for Light microscopy were treated similarly to the NM samples prepared for SEM analyses, as concerns the fixation and dehydration procedures. Afterwards, they were infiltrated with various percentages of LRWhite resin/ethanol mixtures and then embedded in LRWhite resin for 2 days at 50°C. Blocks were cut into semithin sections (1µm) by a Reichert Ultracut ultramicrotome and collected onto slides, stained by Toluidine Blu and observed at a Zeiss (Axiophot) microscope equipped with a colour video camera (Axio Cam MRC) using a computer-assisted image analysis system (AxioVision).

S4.3.2 - TEM Imaging

For TEM imaging plant samples were prepared similarly to those used for Light microscopy until the inclusion in LRWhite resin blocks. The blocks were then cut into ultra-thin sections (60-80 nm) by a Reichert Ultracut ultramicrotome and stained with uranyl acetate and lead citrate. Micrographs were captured by a JEOL 1200 EX II electron microscope equipped with Olympus SIS VELETA CCD and iTEM software.

S5 - Experimental design settings

Two stocks of duckweed cultures were then prepared, after another sterilisation step, by cultivating fronds of *L. minor* L. in plastic vessels for 10 d in 1/10 strength Hoagland's nutrient solution in the presence (Fe-supplied, Stock1) or absence (Fe-deprived, Stock2) of 6 µM Fe(EDTA) (soluble chelated-Fe). After this period (T_0), duckweed colonies from Fe-supplied and Fe-deprived cultures were transferred into 24-well plates (well volume: 2.8 mL, well surface area: 2 cm^2; BD, USA), and each well was filled with 2.5 mL Hoagland's nutrient solution at pH 5.8 ± 0.1, regardless of the specific iron treatment, and 1-2 colonies (1-2 visible fronds each) of *L. minor* were placed therein. In order to investigate the recovery from Fe deficiency, distinct experimental treatments were planned for specific purposes as follows: i) C^+ = duckweeds from Stock1 grown in optimal Fe supply, i.e. where iron was supplied as 6 µM Fe(EDTA) during the entire experiment; this treatment represented the reference control for duckweeds growing in optimal conditions for all of the nutrients throughout the experiment, i.e. without any limitation for iron. ii) C^- = Fe-starved duckweeds from Stock2 further grown in Fe deprivation; this treatment corresponded to the reference control of duckweeds grown in the total absence of Fe throughout the experiment. iii) C_0 = plants from Stock2 then resupplied with 6 µM $FeCl_3$ (insoluble-Fe), that means they were grown under Fe limitation since only very few ions were present in the soluble form because of the very low solubility of Fe^{3+} at pH 5.8; this treatment was the primary reference control for duckweeds added with the CL-NMs; iv) R = Fe-starved duckweeds from Stock2 resupplied with 6 µM Fe(EDTA) (soluble-Fe); this treatment was another reference control for duckweeds added with CL-NMs, because it aimed at discriminating the effectiveness of CL-NMs in resupplying Fe to plants (mediated bioavailability), as compared with more direct iron resupply composed of soluble Fe (promptly bioavailable). v) T = Fe-starved duckweed plants from Stock2 further grown in Fe deprivation, like C^-, and added with catechol-free (control) electrospun NMs (CNMs); this treatment was aimed at testing the

possible toxicity induced in L. minor plants by CNMs and also represented another reference control for duckweeds added with CL-NMs. vi) *A* = full Fe-supplied duckweeds from Stock1 then grown in optimal Fe supply, like C^+, and further added with CNMs; this treatment was focused at testing possible Fe adsorption caused by CNMs. Such event, in fact, might induce inhibition in the plants caused by the reduction in the amount of soluble-Fe. vii) C_1L-NMs = Fe-starved duckweed plants from Stock2 then grown under Fe limitation since resupplied with insoluble $FeCl_3$, like C_0, and further added with C_1L-NMs (C_1 = 5 mM catechol concentration). This treatment was aimed at testing the capacity of C_1L-NMs to provide duckweeds with Fe(III) that could only be made bioavailable upon the release of chelating agents, the Fe(III)-chelation from insoluble sources ($FeCl_3$) and the Fe-chelate absorption by plants). [1] viii) C_2L-NMs = duckweeds grown and treated as in C_1L-NMs but using C_2L-NMs (C_2 = 100 mM catechol concentration) instead of C_1L-NMs. The scope of this treatment was the same as in C_1L-NMs, but employing NMs with a higher content of catechol to test possible dose effects. A diagram of the various treatments of duckweeds included in the experimental plan is reported in Figure S3. Plates with duckweed cultures trialling various treatments were then covered with transparent lids, to minimise evaporation and accidental contamination, and further incubated under the same photoperiod and temperature used for the preparation of stock cultures, and without renewal (static) of the test solutions. The cultures of duckweed plants were incubated until 96 h and analyses were carried out periodically, i.e. at T_0 and after 24, 48, 72 and 96 h. Each treatment was carried out in six replicates (wells).

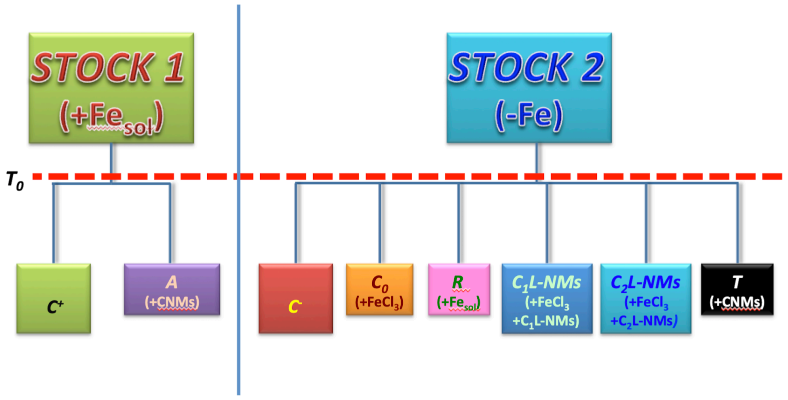

FIGURE S3. Sketch of the experimental plan describing the various Fe-treatments of duckweed plants to test the performances of the distinct catechol-loaded nanomembranes (CL-NMs).

RESULTS

S6 - Chlorophyll fluorescence parameters in duckweed fronds

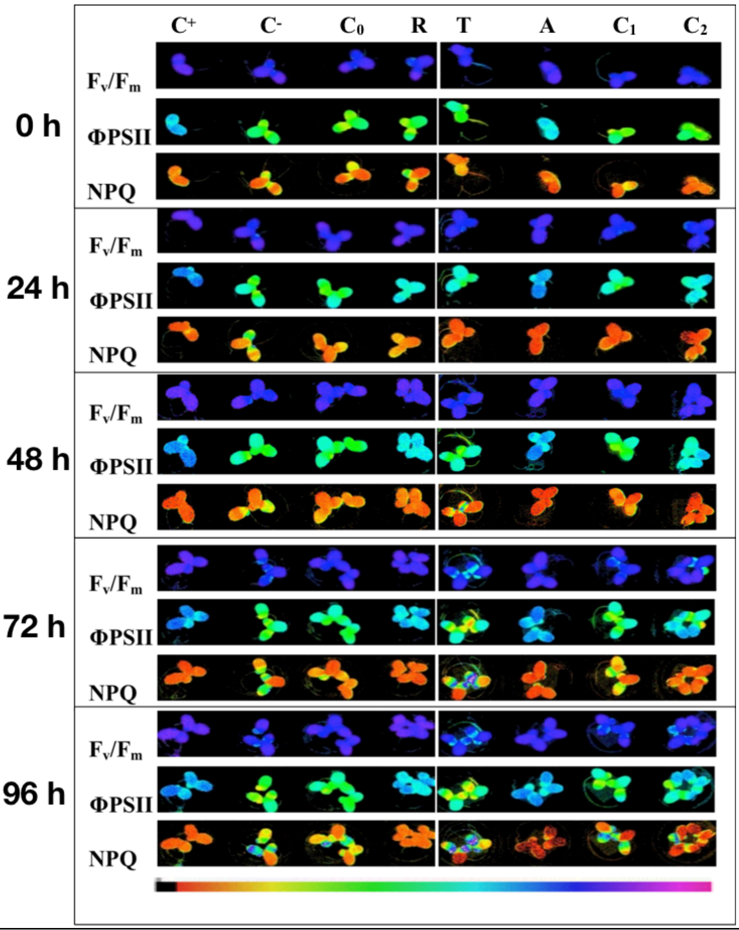

FIGURE S4. Time courses of chlorophyll fluorescence parameters in *Lemna minor* L. fronds treated with different nutrient solutions and electrospun nanofibrous products for (4 days) after T_0. Images of the duckweed plants (captured by an Imaging-PAM M-series system) at actinic illumination of 60 μmol photons m^{-2} s^{-1} after 0, 24, 48, 72 and 96 hours of treatment from T_0 showing: i) F_v/F_m = maximum quantum yield of PSII photochemistry in a dark-adapted leaf, ii) *ΦPSII* = quantum efficiency of PSII photochemistry and iii) *NPQ* = non-photochemical quenching measured at steady-state. The arbitrary colour code depicted at the bottom of each image ranges from 0.000 (black) to 1.000 (pink). Acronyms used for the various treatments are the same as reported in Figure 4 (main text). The image also highlights the presence of an uneven spatial distribution of the photosynthetic efficiency caused by distinct treatments of iron deprivation that can be frequently observed both in different leaves of the same sample and even within each leaf.

S7 - Chlorotic symptoms in duckweed fronds consequent to treatments

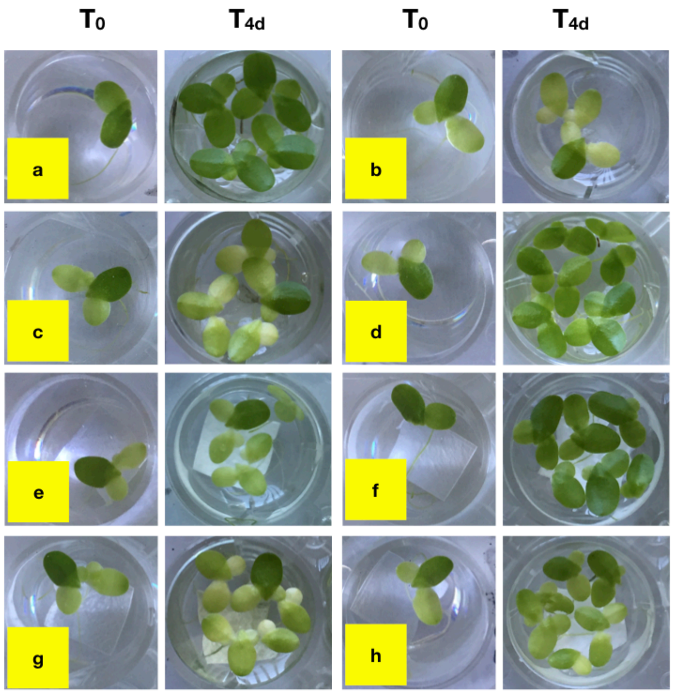

FIGURE S5. Pictures of duckweeds fronds captured before (T_0) and after (T_{4d}) various treatments: a) C^+ = 6 μM Fe(EDTA) for both pre-treatment and treatment; b) C^- = no Fe for both pre-treatment and treatment; c) C_0 = no Fe (pre-treatment) + 6 μM $FeCl_3$; d) R = no Fe (pre-treatment) + 6 μM Fe(EDTA); e) T = no Fe for both pre-treatment and treatment + catechol-free nanomembranes (CNMs); f) A = 6 μM Fe(EDTA) for both pre-treatment and treatment + CNMs; g) C_1L-MNs = no Fe (pre-treatment) + 6 μM $FeCl_3$ + C_1L-MNs (C_1 = 5 mM catechol); h) C_2L-MNs = no Fe (pre-treatment) 6 μM $FeCl_3$ + C_2L-MNs (C_2 = 100 mM catechol).

S8 - Ultrastructural symptoms in leaves and roots consequent to treatments

S8.1 - Fully Fe-supplied control plants (C^+)

In control plants pre-treated and treated with Fe(EDTA) (C^+), leaves observed both by optical microscope and TEM presented several chloroplasts distributed in the mesophyll cells (Figure S6a, light blue arrow). TEM micrographs showed the presence of the internal membrane system of chloroplasts, devoted to photosynthesis typical of low light conditions, i.e. organised in a multitude of thylakoids organised in grana connected by lamellae embedded in a granular stroma [16–18]. Albertsson and Andreasson (2004) reported that thylakoids in grana account for 80% of the global internal membranes of chloroplasts a value that seems to correspond to the amount of thylakoids within grana displayed in TEM micrographs of chloroplasts from leaves of *L. minor* plants grown under C^+ condition [19]. In cells from the same samples, some starch granules were also observed in the chloroplasts (Figure S6a, light blue arrow), as dependent on the light growth conditions [18,20].
Optical microscope observation of roots from C^+-treated *L. minor* plants, for comparison, showed the presence of several chloroplasts, as typically reported in literature, [21] where they were identified in the various cell types surrounding the central tracheary element (Figures S6b,c, red arrowheads) and placed all around the central vacuole (Figures S6b,c, orange arrowheads). The ultrastructural analysis by TEM of root chloroplasts highlighted the presence of some starch granules and plastoglobules in the root chloroplasts of these plants (Figure S6d, light blue and green arrows, respectively). Additionally, several internal membranes could be observed, that differently from leaf chloroplasts were prevalently composed of stromal lamellae (Figure S6d, red arrows) running parallel in the organelle and rarely appressed to form thylakoids and grana (Figure S6d, red dotted arrows). The abundance of photosynthetic organelles and ultrastructures in both fronds and roots confirmed the presence of optimal growth conditions. The PGs present in the root chloroplasts appeared similar to the type described in the leaves, i.e. displaying an inner electron dense core (dark grey) and a surrounding lighter layer (grey) (Figure S6d, green arrow).

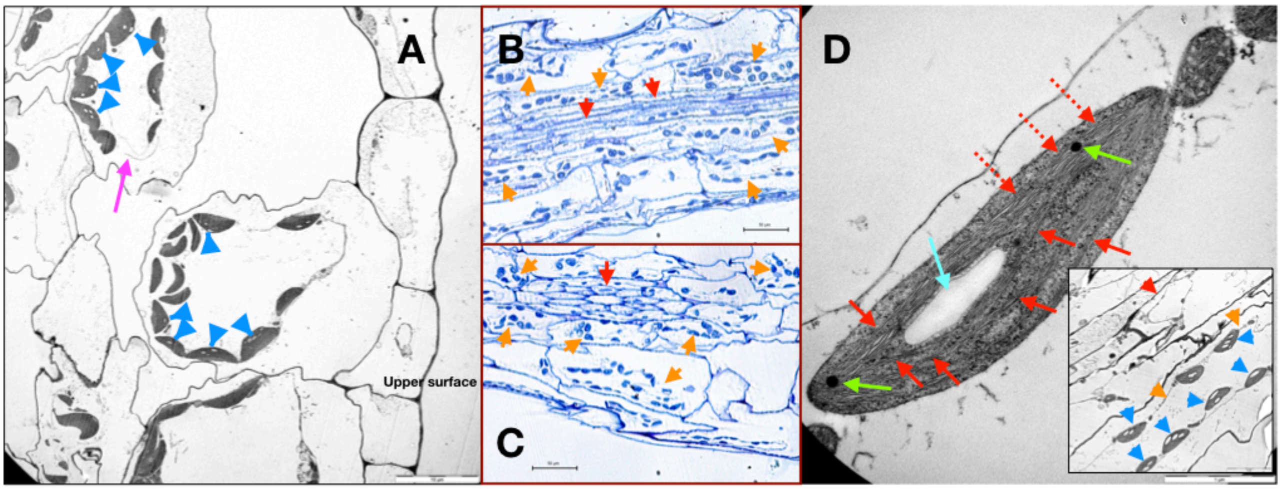

FIGURE S6. Optical microscope images and TEM micrographs of *Lemna minor* L. structures of leaves (**A**) and roots (**B**,**C**,**D**) from C^+ plants: (**A**) cross section of a mesophyll leaf observed by TEM; (**B**) and (**C**) = longitudinal and transversal cross sections, respectively, from duckweed roots observed by optical microscope (x100); red arrows treachery structures; orange arrows chloroplasts in root cells surrounding the treachery structures; (**D**) ultrastructural features of a chloroplast from a C^+ root cell: solid red arrows = double thylakoid stromal lamellae; dotted red arrows = ≥3 thylakoid stromal lamellae; green arrows = Type1 PGs; inset = TEM micrograph of a longitudinal cross section of a C^+ root showing chloroplasts with starch granules (light blue arrowheads) and the treachery structures (red arrowhead).

S8.2 - Fe-deprived plants (C^-)

The C^--treatment seemed to affect remarkably the ultrastructure of duckweed plants, and specifically of chloroplasts [8,9]. Thylakoids in chloroplasts of these cells were strongly influenced by the Fe-deprived growth conditions, resulting in the reduced number of grana per chloroplast and in the number of thylakoids per granum, in TEM micrographs, so that most of the thylakoids were unstacked (see Figure 5b, red arrows, in the main text) [8,9,22]. Furthermore in some cases, the internal membranes of chloroplasts from leaves appeared as inchoate or demolished (Figure S7a), showing an almost complete dismantling of grana ultrastructures, with only faint residual lamellae hardly visible and immersed in the diffused granular and electron dense stroma (Figure S7a, pink arrows) [8,9,23,24]. A similar situation was observed in the C^- root cells, where single thylakoid stromal lamellae were present (Figure S7c red arrowheads), and immature grana consisting of 2-6 thylakoids were displayed (Figure S6c, solid and dotted red arrows). In any case, the value of 20% of the total internal chloroplast membranes accounting for stroma lamellae in chloroplasts by the study of Albertsson and Andreasson (2004) seemed far to be present in this growth condition by both leaves and roots of *L. minor* plants [19]. Furthermore, iron scarcity also reduced the number of starch granules in the mesophyll cells of leaves, relative to C^+ (Figures S7a, pale blue arrows, and S7a inset, light blue arrows) [9,25], although Liping et al. (2013) reported an increase under Fe-limitation [26]. Differently, TEM micrographs of the *C^-*-treated duckweed roots displayed chloroplasts with big starch granules inside (Figure S6b, light blue arrowheads). Moreover, PGs appeared more numerous and larger in C^- than in C^+, in both leaves and roots, maybe because of the stressing effect caused by Fe-deprivation on chloroplasts and of the protecting role of PGs in thylakoids against oxidative stress (Figures S7a,c, green arrows and arrowheads) (see also Figures 5a,b in the main text, for comparison) [9,22,27]. Interestingly, the C^- treatment seemed to induce in the chloroplasts the formation of a variety of different PGs, in terms of size, electron density and morphological shape. Additionally to those reported in the main text in Figure 5b, Figure S7a showed various types of PGs (Type1 and Type2) in leaf chloroplasts. Also in the roots, Type1 (green arrows), Type2 (green arrowheads) were observed. It is worth noting that the various structural shapes and configurations of PGs here observed in the C^- samples (both leaves and roots) have never been reported in the published literature, to our best knowledge, and these might be the first observations supporting the not yet confirmed hypothesis stated by Nacir and Bréhélin (2013) that plastoglobulin (PGL) composition comprising PGs would vary within the PG population of a single chloroplast and that the presence of certain PGLs could define or be related to the specialisation of PGs [28].

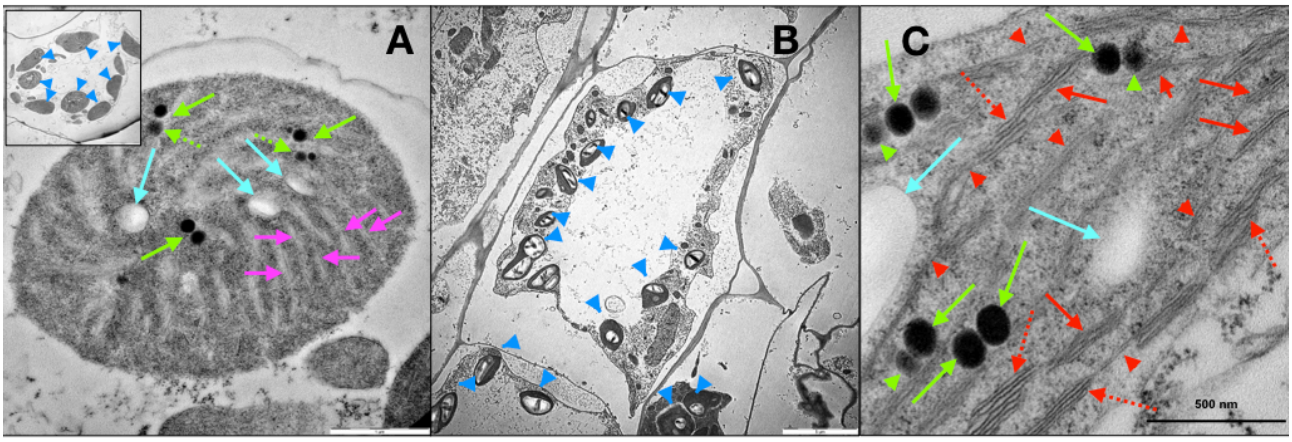

FIGURE S7. TEM micrographs of *Lemna minor* L. structures of leaves (**A**) and roots (**B**,**C**) from Fe-deprived C$^-$ plants: (**A**) TEM micrograph of a mesophyll cell showing chloroplasts with starch granules (pale arrows and inset, light blue arrowheads); solid and dotted green arrows = Type1 and Type2 PGs, respectively; pink arrows = faint thylakoids in stromal lamellae; (**B**) cross section of a duckweed root cell showing starch granules (light blue arrowheads); (**C**) ultrastructural features of a chloroplast from a C$^-$ root cell: red arrowheads = single thylakoid stromal lamellae; solid red arrows = triple thylakoid stromal lamellae; dotted red arrows = ≥3 stacked thylakoids in immature grana; green arrows = Type1 PGs; green arrowheads = Type2 PGs; light blue arrowheads = starch granules.

<u>S8.3 - High catechol-treated plants (C_2L-NMs)</u>

Optical microscope images of *L. minor* leaves and roots from plants treated with *C_2L-NMs* and $FeCl_3$ after the initial Fe-deprivation pre-treatment showed the distribution of chloroplasts typical when plant cells are grown under low-light conditions (here applied), according to the "accumulation movement". [29,30] Starch granules were present in the chloroplasts (Figure S8a, light blue arrows). Cross sections of roots from similar plants of the same treatment, observed by optical microscope, showed that several chloroplasts (Figure S8b,c orange arrowheads) were present in the roots cells surrounding the central tracheary elements (Figure S8b,c red arrowheads). PGs were observed in both leaves and especially in roots (Figures S8d, green arrows and arrowheads). A variety of different PGs, in terms of size, electron density and morphological shape were also present in the roots (Figure S8c) as well as in the leaves (Figure 5c in the main text). Type1 and Type3 PGs were specifically present in the roots (Figure S8d, green arrowheads and green arrows, respectively).

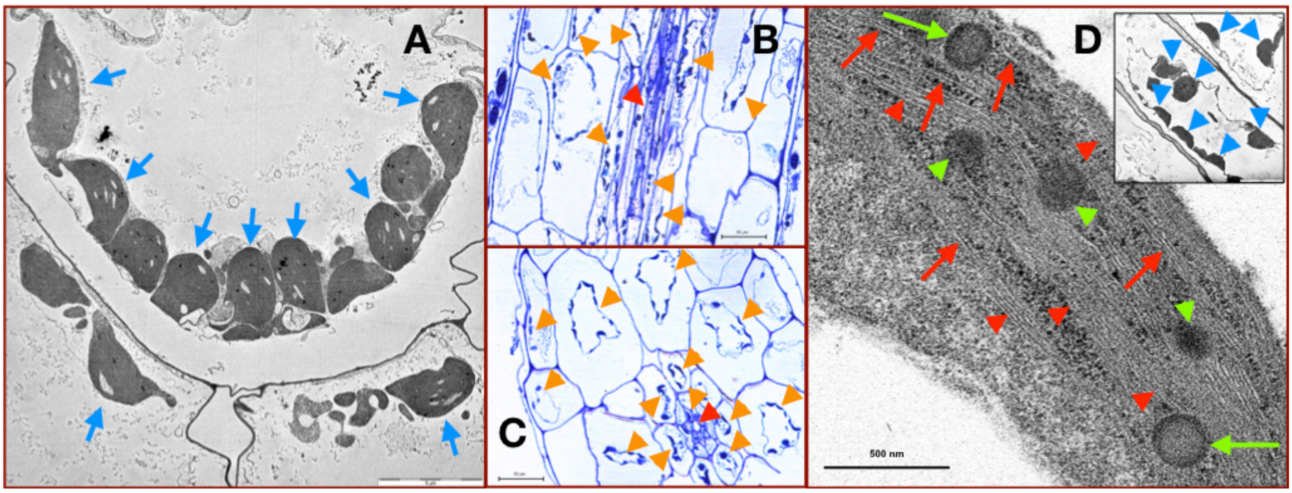

FIGURE S8. Optical microscope images and TEM micrographs of *Lemna minor* L. structures from plants treated with C_2L-NMs and $FeCl_3$ (A = image from a duckweed leave; **B**,**C**,**D** = images from duckweed roots. (**A**) TEM micrograph of a mesophyll leaf: light blue arrows = chloroplasts with starch granules; (**B**,**C**) longitudinal and transversal cross sections of roots observed by optical microscope (x100): red arrowheads treachery structures; orange arrowheads chloroplasts attached to vacuoles in root cells surrounding the treachery structures; (**D**) ultrastructural features of a chloroplast from a C_2L-NM root cell: red arrows = single, double and triple thylakoid stromal lamellae; red arrowheads = ≥3 thylakoid stacked grana; green arrows = Type1 PGs; green arrows = Type3 PGs; inset = TEM micrograph of C_2L-NM root cells showing chloroplasts without starch granules (light blue arrowheads).

REFERENCES

1. Borowski, E. Uptake and transport of iron ions (Fe+2, Fe+3) supplied to roots or leaves in spinach (Spinacia oleracea L.) plants growing under different light conditions. *ACTA Agrobot.* **2013**, *66*, 45–52.
2. Baker, N.R. Chlorophyll Fluorescence: A Probe of Photosynthesis In Vivo. *Annu. Rev. Plant Biol.* **2008**, *59*, 89–113.
3. Gorbe, E.; Calatayud, A. Applications of chlorophyll fluorescence imaging technique in horticultural research: A review. *Sci. Hortic. (Amsterdam).* **2012**, *138*, 24–35.
4. Di Baccio, D.; Pietrini, F.; Bertolotto, P.; Pérez, S.; Barcelò, D.; Zacchini, M.; Donati, E. Response of Lemna gibba L. to high and environmentally relevant concentrations of ibuprofen: Removal, metabolism and morpho-physiological traits for biomonitoring of emerging contaminants. *Sci. Total Environ.* **2017**, *584–585*, 363–373.
5. Pietrini, F.; Bianconi, D.; Massacci, A.; Iannelli, M.A. Combined effects of elevated CO_2 and Cd-contaminated water on growth, photosynthetic response, Cd accumulation and thiolic components status in Lemna minor L. *J. Hazard. Mater.* **2016**, *309*, 77–86.
6. Osório, J.; Osório, M.L.; Correia, P.J.; de Varennes, A.; Pestana, M. Chlorophyll fluorescence imaging as a tool to understand the impact of iron deficiency and resupply on photosynthetic performance of strawberry plants. *Sci. Hortic. (Amsterdam).* **2014**, *165*, 148–155.
7. Dai, Y.; Shen, Z.; Liu, Y.; Wang, L.; Hannaway, D.; Lu, H. Effects of shade treatments on the photosynthetic capacity, chlorophyll fluorescence, and chlorophyll content of Tetrastigma hemsleyanum Diels et Gilg. *Environ. Exp. Bot.* **2009**, *65*, 177–182.
8. Varsano, T.; Kaftan, D.; Pick, U. Effects of iron deficiency on thylakoid membrane structure and composition in the alga Dunaliella salina. *J. Plant Nutr.* **2003**, *26*, 2197–2210.
9. Ladygin, V.G. The effect of root hypoxia and iron deficiency on the photosynthesis, biochemical composition, and structure of pea chloroplasts. *Russ. J. Plant Physiol.* **2004**, *51*, 28–40.
10. Bilger, W.; Björkman, O. Role of the xanthophyll cycle in photoprotection elucidated by measurements of light-induced absorbance changes, fluorescence and photosynthesis in leaves of Hedera canariensis. *Photosynth. Res.* **1990**, *25*, 173–185.
11. Genty, B.; Briantais, J.M.; Baker, N.R. The relationship between the quantum yield of photosynthetic electron transport and quenching of chlorophyll fluorescence. *Biochim. Biophys. Acta - Gen. Subj.* **1989**, *990*, 87–92.
12. Maxwell, K.; Johnson, G. Chlorophyll fluorescence - a practical guide. *J. Exp. Bot.* **2000**, *51*, 659–668.
13. Pietrini, F.; Zacchini, M.; Iori, V.; Pietrosanti, L.; Ferretti, M.; Massacci, A. Spatial distribution of cadmium in leaves and its impact on photosynthesis: Examples of different strategies in willow and poplar clones. *Plant Biol.* **2010**, *12*, 355–363.
14. Evans, J.R. Photosynthetic acclimation and nitrogen partitioning within a lucerne canopy. 2. Stability through time and comparison with a theoretical optimum. *Aust. J. Plant Physiol.* **1993**, *20*, 69–82.
15. OECD Guideline 221: Lemna sp. Growth Inhibition Test. In *Guidelines for the Testing of Chemicals*; OECD Publishing: Paris, 2006 ISBN 9789264016194.
16. Pribil, M.; Labs, M.; Leister, D. Structure and dynamics of thylakoids in land plants. *J. Exp. Bot.* **2014**, *65*, 1955–1972.
17. Castorinis, A. The rotational model: A new hypothesis for thylakoid stacking. *Int. J. Plant Biol.* **2016**, *7*, 5.
18. Lichtenthaler, H.K. Plastoglobuli, Thylakoids, Chloroplast Structure and Development of Plastids. In *Plastid Development in Leaves During Growth and Senescence*; Biswal, B., Krupinska, K., Biswal, U.C., Eds.; Springer Netherlands: Dordrecht, 2013; Vol. 36, pp. 337–361 ISBN 978-94-007-5723-3.
19. Albertsson, P.-A.; Andreasson, E. The constant proportion of grana and stroma lamellae in plant chloroplasts. *Physiol. Plant.* **2004**, *121*, 334–342.
20. Duan, Q.; Jiang, W.; Ding, M.; Lin, Y.; Huang, D. Light affects the chloroplast ultrastructure and post-storage photosynthetic performance of watermelon (Citrullus lanatus) plug seedlings. *PLoS One* **2014**, *9*, e111165.
21. Leng, R.A. *Duckweed: A tiny aquatic plant with enormous potential for agriculture and environment*; FAO: Rome (Italy), 1999;
22. Saito, A.; Shimizu, M.; Nakamura, H.; Maeno, S.; Katase, R.; Miwa, E.; Higuchi, K.; Sonoike, K. Fe deficiency induces phosphorylation and translocation of Lhcb1 in barley thylakoid membranes. *FEBS Lett.* **2014**, *588*, 2042–2048.
23. Thoiron, S.; Pascal, N.; Briat, J. Impact of iron deficiency and iron re-supply during the early stages of vegetative development in maize (Zea mays L.). *Plant, Cell Environ.* **1993**, *20*, 1051–1061.
24. Stocking, C.R. Iron deficiency and the structure and physiology of maize chloroplasts. *Plant Physiol.* **1975**, *55*, 626–31.
25. Chen, L.; Wang, G.; Chen, P.; Zhu, H.; Wang, S.; Ding, Y. Shoot-Root Communication Plays a Key Role in Physiological Alterations of Rice (Oryza sativa) Under Iron Deficiency. *Front. Plant Sci.* **2018**, *9*, 757.
26. Liping, Z.; Hongbo, S.; Xiaohua, L.; Zhaopu, L. Gene Regulation of Iron-Deficiency Responses Is Associated with Carbon Monoxide and Heme Oxydase 1 in Chlamydomonas reinhardtii. *PLoS One* **2013**, *8*, 1–9.
27. Bréhélin, C.; Kessler, F. The plastoglobule: A bag full of lipid biochemistry tricks. *Photochem. Photobiol.* **2008**, *84*, 1388–1394.
28. Nacir, H.; Bréhélin, C. When Proteomics Reveals Unsuspected Roles: The Plastoglobule Example. *Front. Plant*

Sci. **2013**, *4*, 114.
29. Wada, M. Chloroplast movement. *Plant Sci.* **2013**, *210*, 177–182.
30. DeBlasio, S.L.; Luesse, D.L.; Hangarter, R.P. A plant-specific protein essential for blue-light-induced chloroplast movements. *Plant Physiol.* **2005**, *139*, 101–114.
